# Longitudinal survey investigating vectors and reservoirs for *Campylobacter* colonization of chickens on a New Zealand broiler poultry farm

**DOI:** 10.1128/aem.01206-25

**Published:** 2025-08-28

**Authors:** Joanne M. Kingsbury, Nigel French, Anne Midwinter, Rebecca Lucas, Maree Callander, Cody P. Hird, Samantha Smith, Kerry Mulqueen, Roy Biggs, Patrick J. Biggs

**Affiliations:** 1 New Zealand Institute for Public Health and Forensic Science, Christchurch, New Zealand; 2 New Zealand Food Safety Science and Research Centre, Massey University 6420, Palmerston North, New Zealand; 3 Molecular Epidemiology and Veterinary Public Health Laboratory (mEpiLab), Hopkirk Research Institute, School of Veterinary Science, Massey University 169002, Palmerston North, New Zealand; 4 Tegel New Plymouth Laboratory, Tegel Foods Ltd. 165541 , New Plymouth, New Zealand; 5 Poultry Industry Association of New Zealand (PIANZ) 533908 , Auckland, New Zealand; 6 Biggs Food Consultancy Ltd., Whanganui, New Zealand; 7 School of Food Technology and Natural Sciences, Massey University 548347, Palmerston North, New Zealand; Universita degli Studi di Napoli Federico II, Portici, Italy

**Keywords:** *Campylobacter*, reservoir, transmission route, contamination source, meat chicken, chicken colonization, primary production, biosecurity

## Abstract

This longitudinal survey followed the life cycle of a New Zealand broiler flock to investigate sources of flock colonization by *Campylobacter*. Samples were collected at frequent intervals from potential *Campylobacter* reservoirs and sources, transmission routes for *Campylobacter* ingress into the broiler shed, and to monitor flock colonization. Of the 738 samples, 200 (27%) tested positive for *Campylobacter. Campylobacter* species from sample isolates included 316 *Campylobacter jejuni*, 39 *Campylobacter coli,* and 8 *Campylobacter lari* isolates; only *C. jejuni* was isolated from chickens. *C. jejuni* isolates (*n* = 199) were sequenced and consisted of seven sequence types (STs); the most abundant was ST6964 (105 isolates). Most flock isolates were ST6964 (44 isolates) or ST50 (27 isolates). ST6964 isolates closely matched those from the previous flock and another age-matched flock on the same farm, supporting a role for an on-farm reservoir contaminating flocks. There were six STs from catching crew and equipment isolates; the most prevalent were ST6964 (19 isolates) and ST50 (21 isolates). The close genetic match, high *Campylobacter* prevalence in catching samples (59/130, 45%), and the timing of flock colonization occurring closely following catcher presence in the shed support that catchers and equipment might also contaminate the shed and flock from prior flocks that they visited. There was no evidence for wildlife, feed, drinking water, breeder flock, or shed litter as sources of the *Campylobacter* genotypes colonizing the flock. Taken together, this study identified key areas where the poultry industry might focus on-farm risk management practices to reduce colonization of broiler flocks by *Campylobacter*.

**IMPORTANCE:**

Campylobacteriosis is the most frequently notified enteric disease in New Zealand, and New Zealand has one of the highest rates of campylobacteriosis among industrialized countries. Reducing *Campylobacter* colonization of poultry at the farm level would reduce reliance on processing interventions for reducing *Campylobacter* contamination of broiler meat. This study aimed to identify on-farm sources of *Campylobacter* contamination in New Zealand broiler chicken flocks. No evidence was found that wildlife, chicken feed, drinking water, or parent breeder flocks were contaminating sources. Instead, carryover of *Campylobacter* from the previous flock or other farm flocks, and/or contamination from chicken catching crews and their equipment, may have contributed *Campylobacter* strains that colonized the study flock. These are key areas where the poultry industry might focus on-farm risk management practices to reduce colonization of broiler flocks by *Campylobacter*.

## INTRODUCTION

Campylobacteriosis, primarily caused by the species *Campylobacter jejuni* and *Campylobacter coli*, is the most frequently notified foodborne disease in New Zealand, which has one of the highest rates of campylobacteriosis in the industrialized world (1). In 2006–2008, the poultry industry implemented additional biosecurity interventions and changes to slaughter and processing practices which, together with the implementation of the New Zealand Ministry for Primary Industries’ *Campylobacter* Risk Management Strategy, led to a ~50% reduction in the incidence of campylobacteriosis (2). Incidence has continued to decline (albeit more gradually) since 2008 (1), despite an increase in poultry consumption per capita (3). The prevalence and concentrations of *Campylobacter* on carcasses at the end of primary processing have also reduced since 2008, as monitored by the National Microbiological Database (NMD) Programme (4). Despite the improvements, poultry remains the most important vehicle for human infection in New Zealand, estimated to be the source of 84% of campylobacteriosis cases (5). New Zealand Food Safety has set a goal for reducing the number of human cases of foodborne campylobacteriosis by 20% from 88 to 70 per 100,000 population by the end of 2024 (6).

Broiler poultry are typically detectably colonized with *Campylobacter* after 3 weeks of age (7, 8). Once *Campylobacter* enters the flock, fecal contamination of the shed environment from colonized birds facilitates rapid bird-to-bird transmission and colonization of the remainder of the birds within a few days (9). Colonized chickens carry a very high load of *Campylobacter*; for example, concentrations as high as 8 log_10_ CFU/g of cecal contents and 6–7 log_10_ CFU/g of feces have been reported (10–12). Once colonized, on-farm treatments such as bacteriocins, probiotics, feed additives, or bacteriophage treatments may reduce but not eliminate *Campylobacter* loads, and the efficacy varies between flocks and studies (13–15). Current New Zealand poultry processing procedures reduce concentrations present on carcasses by as much as 5–6 log_10_ (16), but contamination of *Campylobacter* between carcasses from colonized birds is likely to occur during processing, and any *Campylobacter* present on carcasses at the end of processing would result in contaminated poultry meat reaching retail. Preventing, reducing the prevalence, or delaying broiler colonization on-farm would minimize the introduction of *Campylobacter* into the processing lines, thereby reducing the risk to consumers. A European Food Safety Authority model has estimated that a 3-log_10_ reduction in broiler cecal *Campylobacter* concentrations would reduce the European Union’s relative risk of human campylobacteriosis attributable to broiler meat by 58% (7). This approach has been successful in reducing the prevalence of *Salmonella* in New Zealand broiler flocks to minimal levels but has been found to be more challenging for *Campylobacter* (17).

Various studies have investigated on-farm risk factors for *Campylobacter* colonization of broiler flocks (7, 9, 18–26). There is less information available about the relative importance of different flock colonization routes, but in recent years, the growing use of highly discriminatory genomic analyses within studies is providing greater potential to identify contaminating sources. There may be multiple important reservoirs, pathways, and risk factors, which may differ from farm to farm, making controlling *Campylobacter* in flocks challenging. Due to the speed with which *Campylobacter* spreads within a shed once present, one biosecurity lapse may be sufficient for *Campylobacter* to colonize an entire flock. Important risk factors have been identified in recent assessments (7, 9, 18–20, 27). Worker movement into the sheds is considered one of the most important transmission routes. There is an increased risk of *Campylobacter* contamination when there has been a *Campylobacter-*positive flock in a broiler house on the farm and when there are neighboring broiler farms. Other nearby livestock, wildlife, pets, and insects can be vectors, but the direction of transmission between these animals and the poultry is not always clear. However, there is limited evidence for transmission from the breeder flock, feed, air, litter, or drinking water (although contaminated standing water around the farm or biofilms in the shed drinking water system might be a problem).

The aim of this study was to provide a better understanding of on-farm sources of *Campylobacter* and routes of transmission into broiler flocks raised in New Zealand. The study involved a longitudinal farm-based microbiological survey testing multiple sample types. These included sampling potential reservoirs from the external environment (e.g., soil, and wild bird and animal feces), potential vectors for *Campylobacter* ingress into the broiler shed (e.g., the breeder flock, insects, rodents, farm workers, and catching crews and equipment), shed inputs (e.g., litter, feed, and drinking water), and testing for *Campylobacter* colonization of flocks (e.g., feces, cecal contents, and cloacal swabs). *Campylobacter* isolated from different sources were then compared with strains colonizing the flock using whole-genome sequencing (WGS) to provide linkages between *Campylobacter* from birds and contaminating vectors and reservoirs. Temporality was also assessed by frequent sampling intervals. Finally, genotypes found on the farm were followed to see which ones survived primary processing and which might be relevant from a public health perspective.

## MATERIALS AND METHODS

### Farm selection, sampling event logistics, and capture of farm variables

Farm selection was based on proximity to the testing laboratory. The farm was owned by the largest poultry company operating in New Zealand, and the shed design, equipment, and operating practices were representative of other farms owned by the same company. The farm was also company-managed (rather than contracted), which enabled easier access for sampling. Poultry-raising shed K2 was selected as the main sampling shed because it had matching parameters to another shed that was stocked at the same time, from which samples were also taken to check for flock colonization (control shed K3). The study and control flocks were sourced from the standard industry breeder flock and hatchery for that farm. The breeder production farm is one of 10 breeder farms in the region that supplies the hatchery, which in turn supplies 30 broiler farms in the region, and sometimes to farms in neighboring regions. The breeder raising shed was sampled during the rearing of the breeders that laid eggs for the study flock, and the breeder production shed was sampled at the specific times that the eggs were laid and hatched. The catching company used for the different harvests of the flocks in this study catches chickens from multiple farms owned by the same parent company and also farms owned by a second major poultry industry company. The processing plant where cecal contents and carcass rinsates were collected from the study and control flocks was also the same industry plant typically used by the broiler farm. The entire survey was conducted from July to December 2019; the rearing period of the selected flock was timed to coincide with peak prevalence of *Campylobacter* in poultry, which occurs during the austral summer months (as defined by industry NMD Programme prevalence data).

To inform variables that might influence *Campylobacter* ingress into sheds, data were collected regarding on-farm variables, staff, potential risk factors associated with *Campylobacter,* and biosecurity measures (Table S1). In addition, all workers accessing the shed were given a diary with the request to document any occurrences or observations in the shed of an event different from the norm (for example, observations of wild birds or rodents in the shed, equipment malfunction, or changes in shed activities; captured in Table S4). Weather variables were also collected over the sampling period (Fig. S1).

Typically, the same subset of samplers attended each sampling event and included an industry expert and two trained assistants. All cloacal swabbing was conducted by an industry veterinarian. Assistance was provided by the farm manager and farm workers, who were also trained in sampling techniques and logistics. Aspects of training included standardization of sampling for each sample type, sampling order (sampling sample types with a low risk of contamination first and finishing with sample types with the highest risk of contamination), sterile technique, changing gloves between sample types, sample labeling, and sample sheet documentation.

### Sample types, sampling procedures, and sampling timeline

While there are recommended methods available for testing some of the samples of interest (for example, chicken carcass rinsates, cecal material, and feces), methods for collecting and testing other samples were highly variable. Before the survey commenced, a pilot study tested the suitability of a selection of sample types and proposed test methods (elasticized hair covers in maximum recovery diluent [MRD] for use as boot socks, 3M Enviro-swabs, houseflies with and without flypaper, and unused litter consisting of wood shavings with and without MRD) inoculated with different concentrations of a cocktail of 12 *C*. *jejuni* and *C. coli* strains. These sample types were selected because they were not routinely tested by the Tegel New Plymouth laboratory, were diverse, or contained competing microbiota or inhibitors that might affect downstream analyses. Methods and results are summarized in the supplemental material. Of the samples tested, the best detection of *Campylobacter* was from inoculated house flies with or without flypaper (thus, glue traps present on the flypaper were non-inhibitory), followed by swabs and hair covers in MRD, while *Campylobacter* was only isolated from litter with MRD, at the highest level of inoculum. *C. coli* and *C. jejuni* were isolated from each sample type. Taken together, the sampling and testing methods were deemed suitable for inclusion in the farm survey.

Sample types, numbers, and sampling methodology were informed by other studies. Table S2 provides a detailed description of the sample types tested in the farm survey, numbers of samples of each type collected, methodology for each sample type, rationale for testing each sample type, and time points at which samples were collected. In general, sample selection rationale was based on whether samples were potential vectors for carrying *Campylobacter* into the shed, potential reservoirs or sources of *Campylobacter*, or provided evidence of flock colonization with *Campylobacter*. Potential vectors and reservoirs were further categorized according to whether they were isolated from the internal shed or external environment, catching crew, and equipment. Isolates colonizing chickens were categorized as being from the study flock, the previous flock from the same shed, or the control flock.

The timing for sampling different sample types was as follows: sampling of the breeder flock and environment using boot socks occurred at the breeder rearing farm and laying shed preplacement of the breeder flock into the laying shed, 2 weeks following transfer of breeder birds into the laying shed, and during the time of egg collection for the study and control flocks (120, 99, and 25 days prior to hatching and placement of the study flock, respectively). The broiler raising shed (K2) was sampled at depopulation of the previous flock (boot socks and ceca; 19 days prior to placement of the study flock). Sampling was also conducted post-cleaning, sanitation, and drying, before and after litter placement (boot socks, swabs of floor cracks and potential harborage sites, annex, nipple drinkers, fans, vents, heater duct, feed entry, and drinking water), and 7 and 5 days before placement of the study flock, respectively. Samples of the paper lining of chick crates and swabs of chick transport equipment were taken at the time of placement of newly hatched chicks. Samples external to the shed (boot socks of soil, swabs of wild bird and rabbit droppings, worker aprons, and feed) and inside the shed (crawling insects, boot socks, drinker swabs, fly papers, swabs of annex and nipple drinking lines, and drinking water) were taken at approximately five-day intervals (at flock ages of 5, 10, 15, 20, 25, 28, 35, and 40 days). At the same time, cloacal swabs of chickens were taken to ascertain colonization. Thinning (harvests or depopulations) occurred when the study flock was 29, 36, and 41 days old, and the control flock was 30, 35, and 38 days old. During thinning, swab samples were collected from the catching crew’s clothing (boots and gloves used during the catching of prior flock) and equipment (chicken crates, modules into which crates are placed, forklift that carries modules, chicken transport truck curtain and cab, and catcher-transporting van wheels and cab). For each thinning cut, cecal contents and rinsate samples from carcasses following primary processing were collected at the processing plant. Cecal samples were also taken from the study and control flocks aged 15, 20, and 35 days from chickens that had been culled for routine coccidiosis testing. Additional sample types included swabs of the trucks entering the property (steering wheel, foot pad and/or wheel arches of delivery of feed, litter and LPG, and electrician) and gut contents of wild birds found dead. Following collection, samples were placed into a cooler on ice packs and transported to the testing laboratory.

### Laboratory processing and isolation of *Campylobacter*

Laboratory testing of all samples occurred within 24 hours of sampling. Where possible, samples were tested by the Tegel New Plymouth Laboratory on the day of receipt. For samples that arrived too late in the day to initiate testing, all sample documentation and processing occurred upon receipt by the testing laboratory, samples were stored at 4°C, and sample enrichment commenced the following morning. Specific details for the processing and enrichment of each sample type are provided in Table S3.

All media were sourced from Fort Richard Laboratories, Auckland, New Zealand. All samples were enriched using the same enrichment broth (Bolton broth) and incubated at the same temperatures and for the same duration to ensure consistency and improve the recovery of *Campylobacter* present in low concentrations or under stress. Cecal contents and chicken carcass rinsates were also plated directly onto modified charcoal cefoperazone deoxycholate agar (mCCDA). Bolton broth volumes were typically added at a ratio of one part sample to nine parts Bolton broth; for example, a volume of 90 mL Bolton broth was added to a 10 g poultry sample. All plates and enrichment broths were incubated under a microaerobic atmosphere created using CampyGen gas sachets (2.5%–9.5% CO_2_ and 6.2%–13.2% O_2_; Oxoid, ThermoFisher, Waltham, MA, USA) in an airtight container. Enrichment broths were incubated at 35°C for 4 hours, followed by 42°C for 44 hours. Following enrichment, a 10 µL volume of the enrichment culture was plated onto mCCDA plates. Plates were incubated at 42°C and examined after 48 ± 2 hours for the presence of suspect *Campylobacter* colonies. Individual suspect colonies were streaked onto Columbia Horse Blood Agar plates and incubated at 42°C for 48 hours. Isolates were confirmed as *Campylobacter* spp. via *Campylobacter* latex agglutination (Ngaio *Campylobacter* Latex, Ngaio Diagnostics, Nelson, New Zealand) and by the detection of oxidase activity (Microbact oxidase strips, Oxoid, Hampshire, UK).

Where colonies were present, up to four colonies per positive sample of suspected *Campylobacter* spp. were purified and swabbed onto Amies Transport Medium with charcoal (Copan, Brescia, Italy) and shipped chilled to *^m^*EpiLab, Massey University. The species of up to two isolates per sample was identified using matrix-assisted laser desorption ionization-time of flight mass spectrometry with the extended direct transfer method in a MicroFlex Bruker Biotype (28). Isolates were stored at −80°C for future analysis.

### Whole-genome sequencing and sequence analysis of *Campylobacter* isolates

Genomic DNA was extracted from pure *C. jejuni* isolate cultures using the QIAamp DNA Mini Kit (Qiagen, Hilden, Germany). DNA was quantified and quality-checked with a Qubit assay (Life Technologies, Oregon, USA). Libraries were prepared from the normalized DNA using the Illumina Nextera XT library preparation kit (Illumina #FC-131-1096). The pooled library was sent to the Massey Genome Service (Massey University, Palmerston North, New Zealand) for quality control checks and storage using the DNAstable Tube Kit (Biomatrica, product code: 93021-001). The library was then sent to NovogeneAIT Genomic (Singapore) for sequencing (2 × 150 base PE reads).

Isolate sequence data were analyzed using Nullarbor2 (29), with default parameters unless otherwise stated. Reads were trimmed with Trimmomatic version 0.36 (30) and assembled with SKESA version 2.3.0 (31). The multilocus sequence type (MLST) scheme was auto-selected with mlst version 2.16.1, and their taxonomy was analyzed with the centrifuge version 1.0.4 module. The seven-loci MLSTs were inferred from the generated contigs using Nullarbor2 version 2.0.20181010. Between-isolate comparisons of the WGS data were analyzed by core genome MLST using outputs from Nullarbor2, whole genome MLST using Fast-GeP version 1.0.2 (32), and single nucleotide polymorphism (SNP) analysis using the Snippy version 4.3.6 output from Nullarbor2. The reads were mapped on a per-isolate basis, and then the mapping results were compared to generate the core SNPs detectable across the isolates in each of the data sets. Further visualizations were performed with different metadata categories. Circular dendrograms were generated using iToL (33, 34). The reference genome for SNP analysis of all isolate genomes was *C. jejuni* RM1221 (ST354) (35), while *C. jejuni* 15AR0984 (ST6964) (36) was used for SNP analysis of the ST6964 genomes only; both strains were from the same CC354 clonal complex. The presence of antimicrobial resistance genes (the resistome) was determined using the data from Nullarbor2. In addition, point mutations from a subset of genes known to confer antimicrobial resistance (23S rRNA and *gyrA*) were searched using a local command-line version of ResFinder 4.0 (37). A customized BLAST search available at https://github.com/dw974/dnatools was used to search genomes for the plasmid-associated gene *traC*.

The generated contigs were also analyzed using Roary version 3.13.0 (38) to generate a pangenome absence/presence matrix using default parameters and the addition of “-e --mafft -r” to generate a core genome alignment using mafft and plots using R, respectively. Subsequent analyses of the pangenome were performed using R version 4.3.2 packages Pagoo version 0.3.17 (39), phangorn version 2.12.1 (40), and rhierBAPS version 1.1.4 (41). The three input data sets were the gff files, the absence/presence matrix from Roary, and a metadata file. The input gff files were imported into Pagoo using the “roary_2_pagoo” function. The data were analyzed to allow visualization of the pangenome using methods described in the Pagoo protocol (39) to generate a multi-panel image.

The core genome phylogeny and population structure of the data set were analyzed using methods described in the Pagoo protocol (39) using phangorn and rhierBAPS with the addition of a midpoint root for the phylogeny. Neutral core gene clusters using a computed Tajima’s *D* value of either ≥2 or ≤−2 were used in the phylogeny, and 10 populations were used for the rhierBAPS lineage analysis. A combined figure was drawn of the computed tree showing the lineages and STs.

The genomes of ST6964 isolates from this study (*n* = 104) were similarly compared with ST6964 genomes from other studies. These data sets included genomes from isolates collected from New Zealand poultry carcasses and human cases of campylobacteriosis, including 230 from 2014 to 2016 (36) and 64 from 2019 (5), as well as three isolates from human stool samples from Switzerland and another from a human sample from Denmark (downloaded from PubMLST; https://pubmlst.org/).

## RESULTS

### Overview of farm and flock variables

An overview of farm variables is provided in Table S1. The broiler farm involved in the study had nine raising sheds. Relevant to the transmission of *Campylobacter* on and around the farm, there were no pets or other livestock present on the farm during the study period, but wild birds, rabbits, and rodents were present. The adjacent farm (~100 m away at the closest point) contained dairy cows (but not calves), and the nearest poultry farm was ~3 km away. Biosecurity protocols in operation at the farm included a 36-hour standdown between non-company visitors visiting other poultry farms. Staff were not permitted to keep any avian species at their residences. Rodent bait boxes were present outside each shed, but there were no fly traps. Staff were trained on biosecurity aspects such as understanding *Campylobacter* transmission and ecology and minimizing flock colonization.

**Fig 1:**
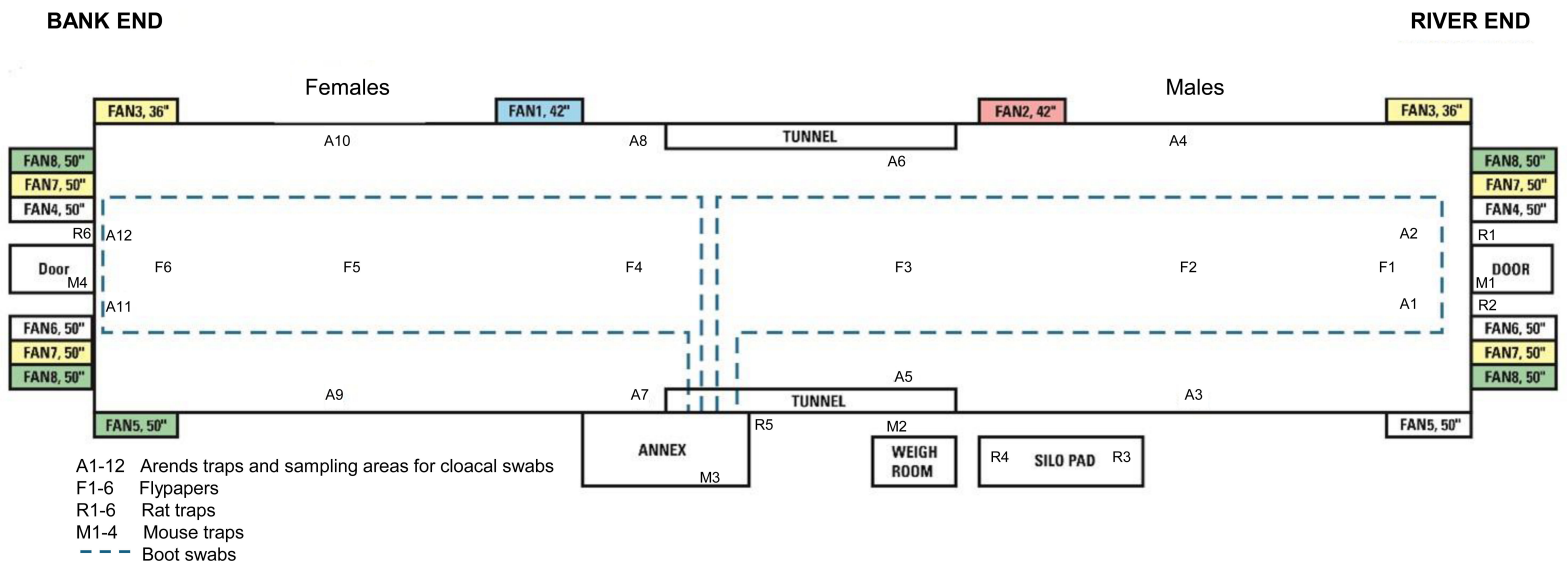
Map of shed K2 (133 m long by 16 m wide), which housed the main flock sampled in this study. The locations of sampling sites are indicated. Shed K3, which housed the control flock in the study, had a similar layout.

Shed K2 that housed the study flock (Fig. 1) was built in 2014 and was 133 m in length and 16 m in width, holding up to 40,000 chicks at placement. Construction included a concrete floor and nib walls surmounted by an insulated, steel-faced panel. Temperature, humidity, and ventilation control were all automated, and ventilation was by extraction fans on either end of the building and vents down each side (136 vents in total). Access was through an annex where hands were washed, sanitized, and boots changed on entry to the shed. The personnel pathway outside the shed was gravel, and there were concrete pads at the shed ends. Male and female birds were separated, with male birds at the river end of the shed and females at the bank end. The control shed designated K3 was a mirror image of K2. The chicks for both sheds came from the same single breeder flock and hatchery and were delivered on the same day; the only difference between the two flocks was the shed in which they were housed.

Feed and water were always available to the birds. The water was sourced from a river and bore and was chlorinated near the source and then with chlorine dioxide before entry into the shed. The water was dispensed through nipple drinkers. The feed was delivered to the farm by feed tankers into enclosed feed silos, and feed transit from the silo to the shed was also enclosed.

Catching crews and their equipment were typically involved in catching for multiple farms each day. Although gloves were changed between farms, clothes were not typically changed. Boots were cleaned and sanitized between flocks following catching before moving off-farm. The processing plant where all thinning cuts were slaughtered and processed was an industry plant. The processing plant processes multiple flocks per day. Primary processing included multiple physical and chemical decontamination interventions, including high scald temperatures, chlorine spray steps, chlorine immersion chiller, and a final acidified sodium chlorite dip; after which, rinsate samples of carcasses were collected.

### *Campylobacter* sample prevalence and spatiotemporal analysis

In total, 738 samples were tested for *Campylobacter* by cultural isolation; 200 (27%) tested positive. The sample prevalence, stratified by sampling time, location, and type, is detailed in Table 1. For sample categories considered as potential vectors or reservoirs for *Campylobacter* ingress into the broiler shed, the highest *Campylobacter* prevalence was from catchers and catching equipment, with 59 of the 131 samples testing positive (45%), including samples from each thinning cut. *Campylobacter* was isolated from all types of catching samples (gloves, boot swabs, crates, particularly where fecal matter was present, modules, forklift, catcher van wheels and cab). *Campylobacter* was also detected from crawling insects captured in Arends tubes (3 of 33 samples; 9%), all of which were identified as darkling beetles or their larvae (Table S5). Rabbit and wild bird feces external to the broiler shed and a worker apron swab also tested positive (4 of 10 [40%], 4 of 33 [21%], and 1 of 8 [13%] samples, respectively). *Campylobacter* was not detected from any feed (*n* = 15), drinking water (*n* = 18), flying insect/flypaper (*n* = 29), or non-catcher trucks entering the farm (electrician or those transporting feed, litter, or LPG; *n* = 6). The insects captured by the flypaper overwhelmingly consisted of small midges (as many as 41 in one sample), while small flies, moths, and mosquitoes were captured infrequently (Table S5). No rodents were captured during the study, and rodent feces were not observed around traps.

**TABLE 1:** *Campylobacter* sample prevalence stratified by sample category, sample type, and sampling time^*a*^

Flock age (days)	A^*b*^	0	1	5	10	13	15	20	25	28	29	30	34	35	36	37	38	40	41	Total (%)
Breeder flock																				
Breeder rearing and laying shed pre-/post-placement (boot socks)	0/10																			0/10 (0)
Boot socks during egg collection	**4/4**																			**4/4 (100**)
Previous flock																				
Ceca	**10/10**																			**10/10(100**)
Shed environment (boot socks)	**2/2**																			**2/2 (100**)
Control flock																				
Ceca							0/5	0/5		0/5		0/10		**14/15**			**10/10**			**24/50 (48**)
Carcass rinsate												**1/6**		**1/6**			**3/6**			**5/18 (28**)
Shed environment (boot socks)										0/2			**2/2**			**2/2**				**4/6 (67**)
Study flock																				
Chick papers and transport		0/7																		0/7 (0)
Cloacal swab				0/12	0/12		0/12	0/12	0/12	0/12				**12/12**				**12/12**		**24/96 (25**)
Ceca							0/5	0/5		0/5	0/10			**5/5**	**10/10**				**10/10**	**25/50 (50**)
Carcass rinsate											**1/15**				**10/15**				**8/15**	**19/45 (42**)
Study flock internal shed environment and inputs																				
Boot socks	0/2			0/2	0/2		0/2	0/2	0/2	0/2				**2/2**				**2/2**		**4/18 (22**)
Drinker swabs	0/5			0/6	0/6		0/6	0/6	0/6	0/6				0/6				**3/6**		**3/53 (6**)
Drinking water	0/2			0/2	0/2		0/2	0/2	0/2	0/2				0/2				0/2		0/18 (0)
Feed								0/3	0/3	0/3				0/3				0/3		0/15 (0)
Shed swabs	0/22																			0/22 (0)
Annex swabs				0/3	0/4		0/4	0/4	0/4	0/4				**1/5**				**2/5**		**3/33 (9**)
Flying insects (flypaper)					0/2		0/4	0/5	0/5	0/4				0/4				0/5		0/29 (0)
Crawling insects (Arends tubes)				0/3	0/2			0/7	0/8	0/8				**2/3**				**1/2**		**3/33 (9**)
Other (paper)				0/1	0/2															0/3 (0)
External environment																				
Rabbit feces							0/3	**3/3**	0/1	0/1								**1/2**		**4/10 (40**)
Wild bird feces, viscera, nest				0/3	0/4		0/5	**3/9**	0/4^*c*^					0/1				**1/8**		**4/34 (12**)
Soil (boot socks)				0/8				0/8		0/2				**1/8**				**1/1**		**2/27 (7**)
Worker apron (swab)				0/1	0/1		0/1	**1/1**	0/1	0/1				0/1				0/1		**1/8 (13**)
Vehicle swabs (e.g., feed, LPG, electrician)		0/1	0/1			0/1	0/1	0/1	0/1											0/6 (0)
Catching crew and equipment											**12/27**	**16/37**			**14/33**				**17/34**	**59/131 (45**)
Total	16/57 (28)	0/8 (0)	0/1 (0)	0/41 (0)	0/37 (0)	0/1 (0)	0/50 (0)	**7/73 (10**)	0/49 (0)	0/57 (0)	**13/52 (25**)	**17/53 (32**)	**2/2 (100**)	**38/73 (52**)	**34/58 (59**)	**2/2 (100**)	**13/16 (81**)	**23/49 (47**)	**35/59 (59**)	**200/738 (27**)

^*a*^ Numbers are in bold font where there was at least one detection of *Campylobacter* from the sample set.

^*b*^ “A” designates all sampling time points prior to hatching of the flock. Sampling of the breeder flock occurred 120, 99, and 25 days prior to hatching of the study flock. The previous flock was depopulated 19 days prior to hatching, and sampling the shed following cleaning and disinfection occurred 7 days (before litter placement) and 5 days (after litter placement) before placement of the study flock.

^*c*^ One sample was taken the following day (day 26).

Considering temporality, samples from the previous flock at the final cut (cecal samples at primary processing) and the K2 shed environment (boot socks), all tested positive for *Campylobacter*. However, there were no isolations from within the K2 shed following cleaning and disinfection, before (*n* = 22) and after litter placement (*n* = 9). Samples from the study flock (cloacal swabs, *n* = 72, and cecal contents, *n* = 25), as well as environmental samples within the broiler shed (*n* = 143), remained *Campylobacter* culture-negative until after the first cut. At this time, a high proportion of catching crew and equipment samples tested positive (12/27, 44% at first cut; and 59/130, 45% of total catching samples). At the subsequent sampling event (flock age of 35 days), a high proportion of chicken samples (cloacal swabs, cecal contents, and carcass rinsates) and shed samples (annex, boot socks, surface and drinker swabs, crawling insects, and nipple drinkers) tested positive for *Campylobacter*. The same was observed for the control flock. Note that there was a single positive chicken carcass rinsate from the first cut of both the study and control flocks, which could have come from either the flock itself or carcass contamination from other flocks processed on the same day.

The species was determined for up to two isolates per positive sample; results for the different sample categories are shown in Table 2. The species breakdown included 316 *C*. *jejuni*, 39 *C*. *coli,* and 8 *C. lari* isolates. Only *C. lari* was isolated from the breeder flock at the time of egg laying for the study flock, and this species was not isolated from any other samples. *C. coli* was isolated from catchers and catching equipment at the first and second cuts of the study flock (18 isolates from nine samples) but was not isolated from the study flock or the K2 shed environment. *C. coli* was also isolated from catching samples during the first cut of the control flock (flock age: 30 days; 11 isolates from six samples), from control boot socks of the control flock K3 shed environment at 34 and 37 days (six isolates from three samples), and from ceca samples of the control flock the following day at the final cut (four isolates from two samples). All other isolates were *C. jejuni*, which was the only species isolated from the study flock (cloacal swabs and cecal and rinsate samples), K2 shed environmental samples (boot socks, drinker and annex swabs, and crawling insects), and previous flock inhabiting the shed. *C. jejuni* was also isolated from wild bird and rabbit feces and an apron swab on day 20.

**TABLE 2:** *Campylobacter* species per sample category^*a*^

Sample category	Sample type	Total positive samples	*Campylobacter* isolates		
			*C. jejuni*	*C. coli*	*C. lari*
Breeder flock	Boot socks during egg collection	4	0	0	8
Previous flock	Boot socks	2	4	0	0
	Ceca	10	20	0	0
Study flock	Cloacal swabs	24	47	0	0
	Ceca	25	25	0	0
	Carcass rinsate	19	54	0	0
Control flock	Ceca	24	30	4	0
	Carcass rinsate	5	10	0	0
Catching crew and equipment	Study shed K2	44	65	18	0
	Control shed K3	16	20	11	0
External environment	Rabbit and wild bird feces, worker apron	7	13	0	0
Internal environment	Study shed K2: boot socks, drinker swabs, annex swabs, crawling insects	16	26	0	0
	Control shed K3: boot socks	4	2	6	0
Total		200	316	39	8

^*a*^ The *Campylobacter* species was tested for up to two isolates per positive sample.

### Genomic analyses of *C. jejuni* isolates

Although *C. jejuni*, *C. coli,* and *C. lari* were all identified from environmental samples, *C. jejuni* was the only species also isolated from the study flock (cloacal swabs, cecal contents, and carcass rinsates). Because the aim of this study was to understand the reservoirs and vectors for *Campylobacter* colonization of poultry, only *C. jejuni* isolates were selected for whole-genome sequencing. For most positive samples, a single isolate was selected for sequencing. However, two isolates were sequenced from positive samples from early time points, as samples might contain multiple *Campylobacter* types, and the objective was to identify the earliest occurrence of genotypes from reservoirs or vectors into the broiler shed that went on to colonize the flock. A total of 199 *C*. *jejuni* isolates arising from 165 positive samples were sequenced.

The 199 whole-genome-sequenced isolates comprised seven different seven-gene multi-locus sequence type (ST) profiles (Table 3). *C. jejuni* STs included ST6964 (105 isolates), ST50 (60), ST45 (24), ST3105 (4), ST12269 (4), ST25 (1), and ST53 (1). One isolate was identified as a single locus variant of ST6964; while this isolate was considered as ST6964 for this study, it has been assigned a new sequence type (ST12270) from the PubMLST database. The majority of isolates from the study flock were ST6964 (44 isolates), and this ST was also isolated from the previous flock and control flock, catching samples, and the K2 shed environment. ST50 was also isolated from the study flock (27 isolates), control flock (2 isolates), and previous flock (1 isolate), as well as catching samples and the K2 shed environment. There were four additional STs among the catching crew and equipment isolates (ST45, ST3105, ST25, and ST53). Most isolates from rabbit feces, wild birds, and the workers’ aprons were ST45. Four isolates were typed as being single locus variants of ST3663, which was a new allelic profile that had not been previously reported; thus, they were assigned a new PubMLST sequence type (ST12269). Two of the ST12269 isolates were from a single sample of rabbit feces, and the other two from a single sample of the annex boot transit area, all from the same sampling event at a flock age of 40 days.

**TABLE 3:** Multi-locus sequence types of *C. jejuni* isolates stratified by sample category

ST	Isolates from flocks (cloacal swabs, ceca, and carcass rinsates)			Catching crew and equipment	Internal environment (insects, boot socks, annex, and drinkers)	External environment (worker apron, wild bird, and rabbit feces)	Total
	Study flock	Control flock	Previous flock				
6964	44	25	11	19	4	2	105^*a*^
50	27	2	1	21	9	0	60
45	0	0	0	13	2	9	24
3105	3	0	0	1	0	0	4
12269^*b*^	0	0	0	0	2	2	4
25	0	0	0	1	0	0	1
53	0	0	0	1	0	0	1
Total	74	27	12	56	17	13	199

^*a*^ One isolate included in the total was actually a single locus variant of ST6964. Although this isolate has been designated a new MLST designation (ST12270), the isolate was considered as ST6964 for the purposes of the study and clustered with the main lineage of ST6964 isolates. A further isolate was typed as ST6964, but the genome was not included in genomic analyses due to poor quality sequence.

^*b*^ *C. jejuni* ST12269 differed from ST3663 at a single allele and was assigned a new ST by PubMLST.

**Fig 2:**
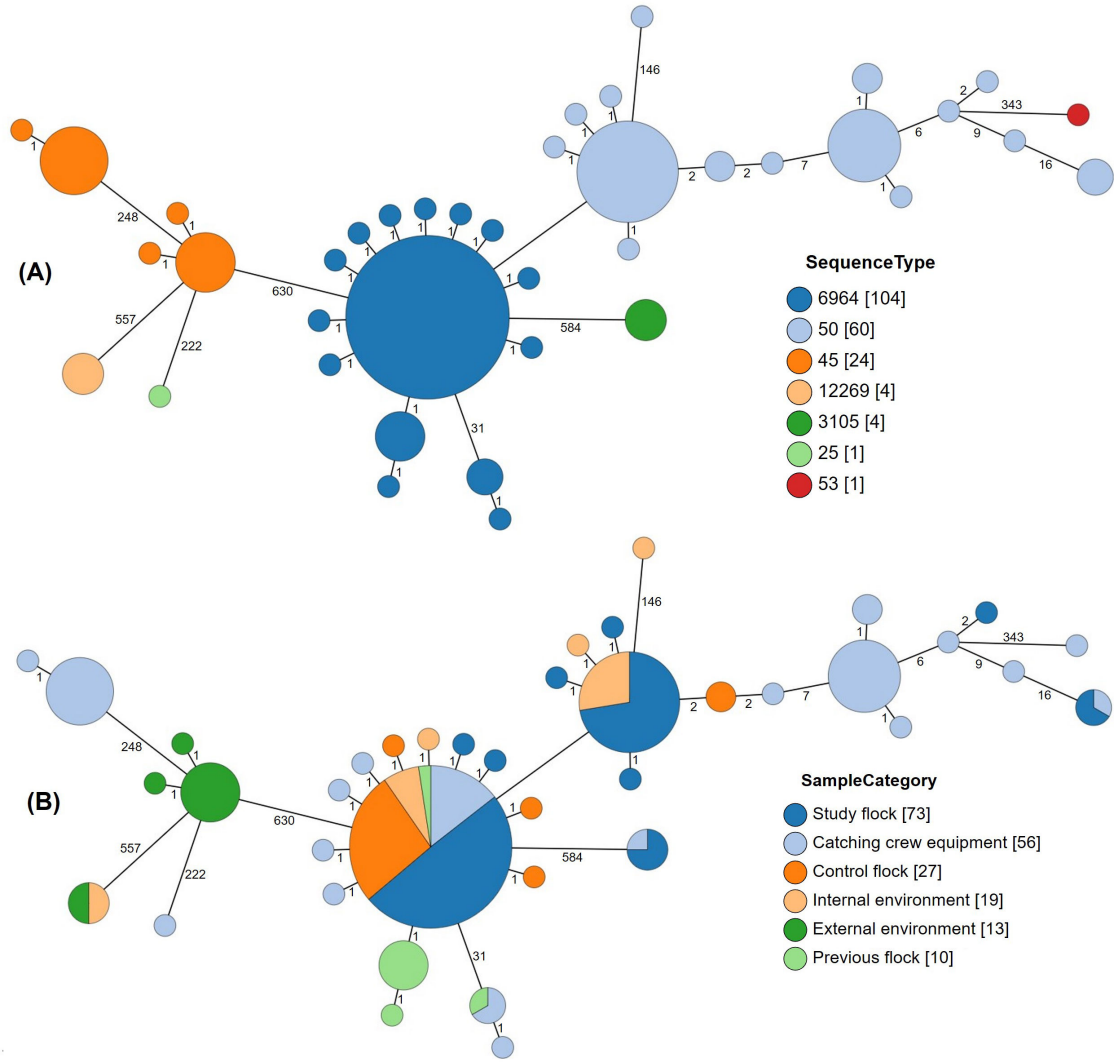
Minimum spanning tree of the *C. jejuni* isolates based on core genome multi-locus sequencing typing profiles, showing the sequence types (**A**), sample categories (**B**), and the number of allele differences between isolates. The size of the circle indicates the number of isolates, and the branch lengths are on a log scale.

**Fig 3:**
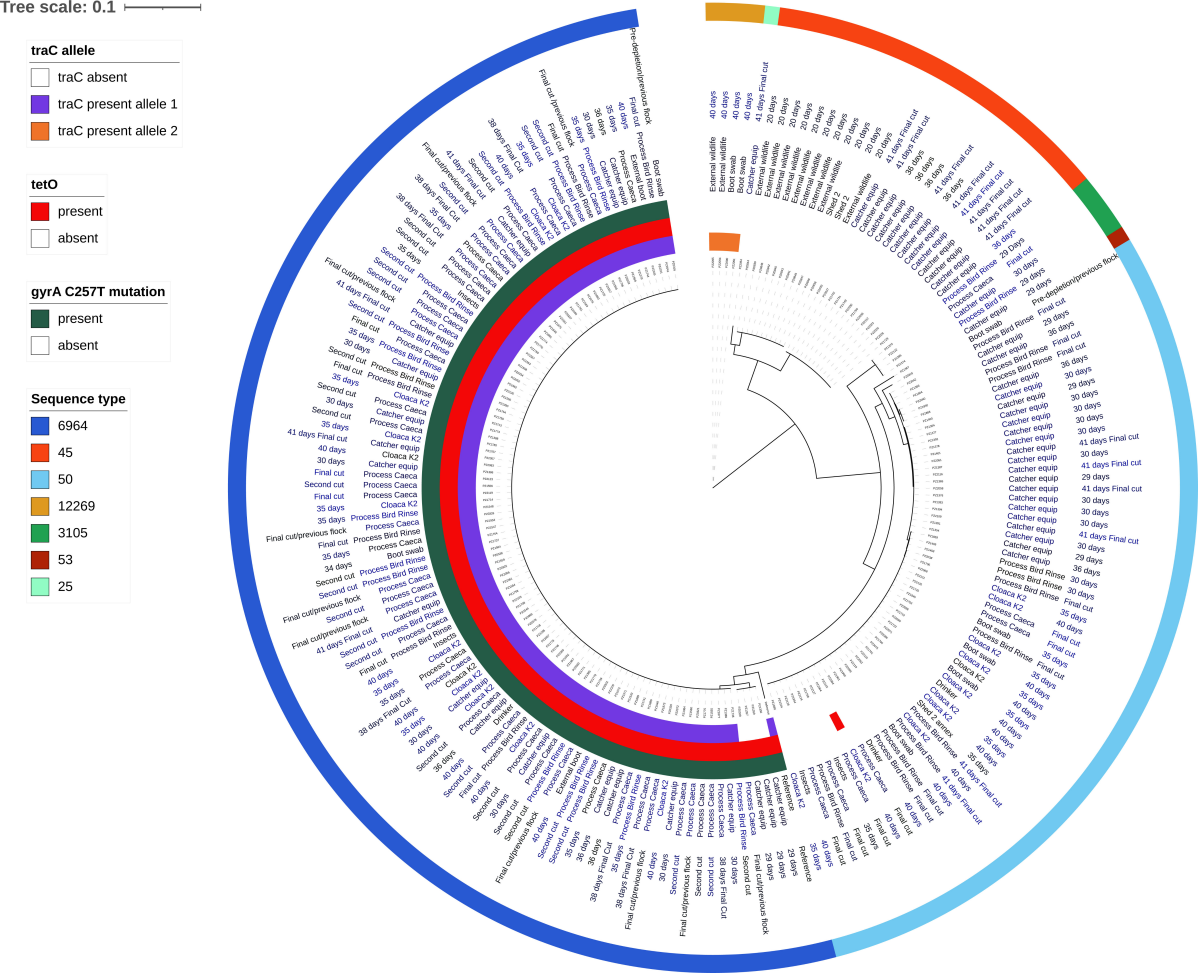
Circular maximum likelihood tree based on single nucleotide polymorphism distance displaying sequence types (outermost band), sample time/cut, and sample type. The colored inner bands depict the presence and allele type of the plasmid-associated *traC* gene, and presence or absence of the *tetO* tetracycline resistance gene and *gyrA* C257T mutation conferring fluoroquinolone resistance. The reference genome was *C. jejuni* RM1221 (ST354).

The genomic data were further analyzed for 198 isolates to link *C. jejuni* isolates from sources, reservoirs, and transmission routes with isolates from colonized chickens (note that although 199 isolates were sequenced, one isolate was removed from further analyses due to low-quality sequence). Visual representations of the genomic data include a minimum spanning tree representation based on core genome multi-locus sequence typing (cgMLST) differences between isolates (Fig. 2), and a circular dendrogram based on single nucleotide polymorphism differences using *C. jejuni* RM1221 (ST354) as the reference genome and with metadata provided for each isolate (Fig. 3). Further genomic analyses compared the pangenomes, core genome phylogeny, and population structure of isolates from the study (Fig. S3 and S4).

There was a high degree of clonality among the *C. jejuni* ST6964 isolates. The majority of isolates differed by 0–1 cgMLST allele, and there was a small cluster that differed from the main cluster by 31 alleles (Fig. 2). The majority of the ST6964 isolates were also indistinguishable via SNP analysis using the *C. jejuni* RM1221 reference genome (zero pairwise differences; Fig. 3). To achieve a finer level of discrimination between isolates, SNP analysis was also performed using a ST6964 reference genome (*C. jejuni* 15AR0984; Fig. S2). A total of 65 isolates (62%) remained indistinguishable in a single monophyletic group (at the top of the plot, starting with PZ0316). These included two cecal and one boot sock isolate from the previous flock and six catcher sample isolates (crate and module isolates from the first cut of the control K3 shed before *Campylobacter* was detected in that flock, and boot swabs and forklift from the second cut of the study flock). Besides two isolates from crawling insects (flock age: 35 and 40 days) and one from the control K3 shed boot socks, all other 53 isolates in this cluster were from either the study or control flock aged 35 days or older (cloacal swabs, cecal contents, and carcass rinsates). A closely related cluster that consisted of seven indistinguishable isolates (e.g., PZ2057) arose from a catcher crate sample at the first cut of the control K3 shed, three cecal or rinsate samples from the second and third cuts from the control shed, and catcher crate and module samples from the final cut of the study shed. Another closely related cluster comprised cecal isolates from the previous flock (bottom of plot, e.g., PZ0338). A further lineage (e.g., PZ0329) was distinct from all other ST6964 isolates and contained a recombinant region; this consisted of an isolate from the previous flock and three catcher module isolates from the first cut of the study flock. Taken together, the earlier detection and close genetic relationships between isolates support that the previous flock and/or catchers and their equipment may have contaminated the study and control flocks.

Several clusters of *C. jejuni* ST50 were present (Fig. 2). One cluster encompassing 17 isolates that differed by 0–1 cgMLST loci was all from catching crew and equipment samples from the first cuts of the control and study flocks and the final cut of the study flock. Another cluster comprised 33 isolates from the study flock (cloacal swabs, cecal contents, and carcass rinsates) and the K2 shed environment (boot socks, crawling insects, and drinker swabs). This cluster was closely related (differing by 3–4 cgMLST loci) to a catcher crate sample from the second cut of this flock and two isolates from a carcass rinsate sample from the first cut of the control flock (differing by 1–2 cgMLST loci). In another cluster, two carcass rinsate isolates from the final cut were indistinguishable from a catcher module sample from the second cut of the study flock. Although ST50 was isolated from the shed environment at depopulation of the previous flock, this isolate differed by at least 146 cgMLST loci from any other isolates in the study. Distinct clusters of ST50 isolates were also shown in the principal component analysis (PCA) representation based on pangenome analyses (Fig. S3C). However, all ST50 isolates form a single lineage together with the ST53 isolate based on rhierBAPS analysis (Fig. S4).

There were also two distinct clusters of *C. jejuni* ST45, which differed by 248 MLST loci (Fig. 2). One cluster of 11 isolates was from rabbit and wild bird feces and worker apron swabs sampled at a flock age of 20 days. The second cluster of 13 isolates was from catching crew and equipment samples from the second and third cuts of the study flock. This ST was not detected from the broiler shed environment, and there was no evidence of flock colonization of this ST (cloacal swabs, cecal contents, or carcass rinsate samples). Consistent with the core genome MLST analysis, two separate lineages of ST45 isolates were also seen in the phylogeny tree based on rhierBAPS analysis (Fig. S4).

### *In silico* analysis of antimicrobial resistance

The 198 *C*. *jejuni* genomes were interrogated for the presence of known antimicrobial resistance genes and alleles (Fig. 3). The only resistance gene identified was *tetO*, which was present in all ST6964 isolates, including the *C. jejuni* RM1221 reference strain, as well as a single ST50 isolate obtained from a cloacal swab from the current flock. In addition, only ST6964 isolates contained the C257T mutation in *gyrA* that results in the T86I functional mutation that confers fluoroquinolone resistance, also found in earlier isolates of this ST (36). No isolates contained the 23S rRNA A2075G allele that confers macrolide resistance.

### Analysis of *C. jejuni* ST6964 lineages

Previously, two major poultry company-specific clades of *C. jejuni* ST6964 were identified that differed by the presence of a plasmid housing a type IV secretion operon that included the virulence determinant *traC* (36). To further characterize the lineage of *C. jejuni* ST6964 isolates present in the flock, genomes were also searched for the presence of *traC*. Isolates within the predominant cluster of *C. jejuni* ST6964 isolates were positive for *traC* (note that the reference strain also contained the plasmid gene). This cluster contained isolates from the previous flock, as well as multiple isolates from birds of the main study flock, and included isolates from carcass rinsates following processing. However, the four isolates within the distinct ST6964 cluster (discussed earlier) were negative for this plasmid gene. The only other ST to contain *traC* was the newly assigned *C. jejuni* ST12269, which had a different allele than the ST6964 isolates. Additional analyses to confirm the plasmid presence were not conducted.

To gain further insights into the population diversification of the *C. jejuni* ST6964 isolates from this study, isolate pangenomes from this study were compared with those from other *C. jejuni* ST6964 isolates from New Zealand poultry and human clinical samples from 2014 to 2016 (230 isolates [2]) and from 2019 (64 isolates [3]), as well as four human clinical isolates from other countries. As represented by PCA plotting, most isolate genomes from this study formed a tight cluster that overlapped with isolate genomes from 2019, while a small number of isolates formed a distinct cluster with other isolates from that study and also clustered closely, but did not overlap, with isolates from 2014 to 2016 (Fig. S5). Phylogenetic analyses placed the 402 ST6964 isolates into three distinct lineages, with the majority of isolates from this study falling within lineage 2, and the remainder within lineage 1 (Fig. S6).

## DISCUSSION

**Fig 4:**
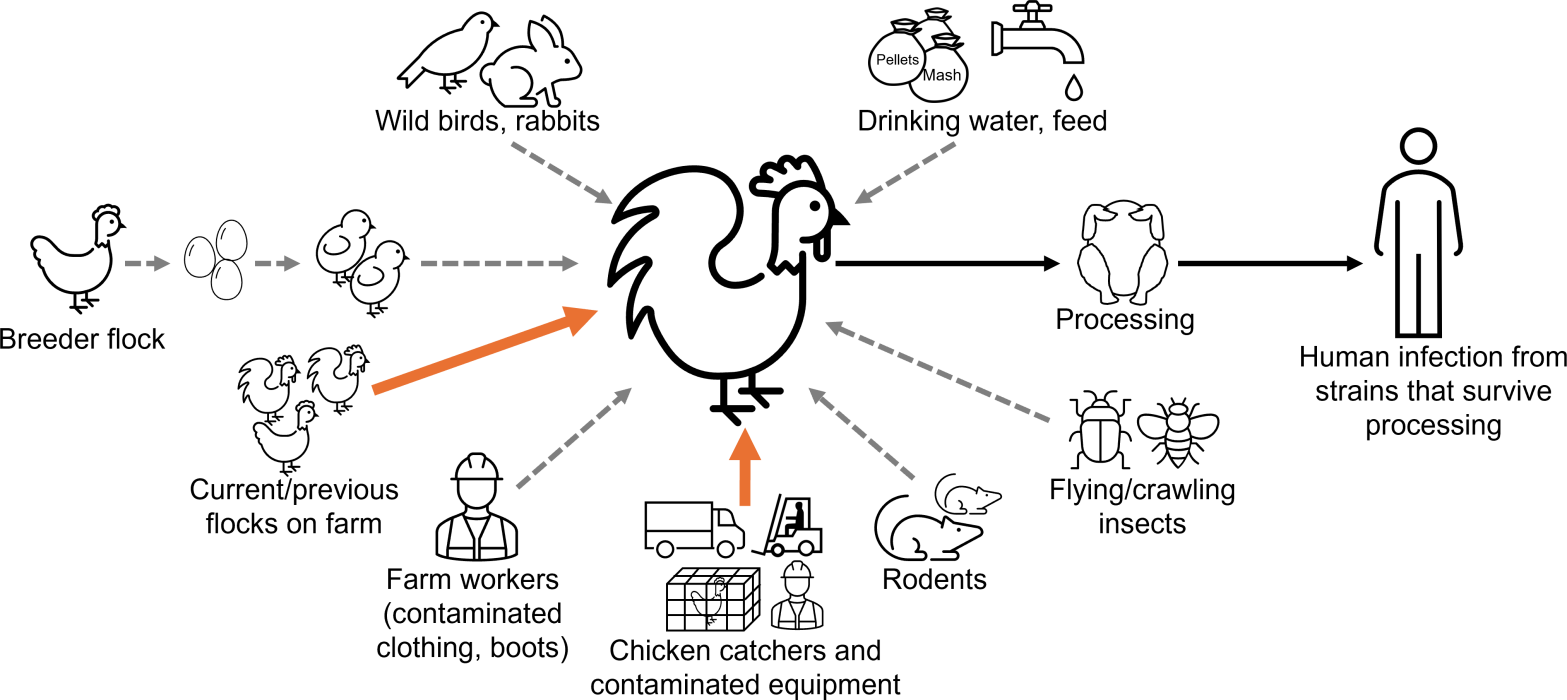
The longitudinal survey tested potential transmission routes and sources for *Campylobacter* colonization of a broiler flock. Roles were found for an on-farm reservoir and catchers and their equipment. The same strains colonizing chickens were also isolated following primary processing and thus may be present in the product at retail and pose a risk for consumers.

The longitudinal survey tested multiple environmental samples for *Campylobacter* from outside and inside the broiler raising shed, catching crew and equipment, and breeder and broiler flock samples over the life cycle of a New Zealand broiler flock. WGS of *Campylobacter* isolates was then implemented to link sources and vectors with the strains colonizing flocks. Isolates from the broiler flock were found to be indistinguishable or closely linked to those from the previous flock and an age-matched flock on the same farm, as well as some isolates from catching equipment, but no linkages were found with isolates from wild birds or animals, the breeder flock, water, or feed. The roles for the different sources and vectors tested in this study are depicted in Fig. 4.

The close genetic linkages between *C. jejuni* ST6964 isolates colonizing the farm flocks support a role for carryover from the previous flock or another on-farm reservoir in contaminating the current flocks. Assessments have determined that a contaminated barn environment due to inadequate disinfection and cleaning between flocks carries a high risk for contaminating the new broiler flock (7, 18). Indeed, a study detected *Campylobacter* from floors, feeders, drinkers, vents, and fans of New Zealand broiler sheds sampled post-cleanout and sanitation, albeit at a lower prevalence and concentration than from samples prior to cleanout (42), highlighting the challenges in completely eliminating *Campylobacter* from sheds between flocks. Interestingly, in the current study, all 31 samples taken from the raising shed post cleanout, sanitation, and drying (swabs from potential harborage sites, water lines and drinkers, water samples, and boot socks following litter addition) tested negative for *Campylobacter*. The results suggest that cleaning and sanitation were effective, but it remains possible that *Campylobacter* was still present in low concentrations or in other protected niches within the shed. *Campylobacter* colonization of chickens under 2–3 weeks of age is rarely detected by culture due, in part, to the protective effect of maternal antibodies, although very low levels of *Campylobacter* DNA have been detected from chicken feces within the first week of age (43–48). However, once present in the shed and initial chicken colonization has occurred, transmission through the flock is rapid (9). Had transmission resulted from flock carryover, flock colonization might have been expected to occur at an earlier age than was observed. Instead, *Campylobacter* was not isolated from any samples from within the shed, and there was no evidence of flock colonization until after the first cut, when the broiler flock was 35 days old. Given that the age-matched control flock was also colonized with indistinguishable strains, the source of the strains might instead include current flocks on the same farm or external unidentified reservoirs such as standing water, with transmission occurring reservoir-to-shed or shed-to-shed on worker clothing, or via other biosecurity breaches. Indeed, *C. jejuni* ST45, which was indistinguishable from rabbit and wild bird feces isolates from outside the shed, was isolated from worker clothing on day 20 (at a stage where the flock remained *Campylobacter*-negative). Furthermore, *C. jejuni* ST50 and ST6964 isolates that matched those from the flock and *C. jejuni* ST12269 isolates that matched those from rabbit feces were also isolated from the shed annex; thus, shed ingress and egress of *Campylobacter* via workers are likely to occur.

Thinning or partial depopulation is a practice whereby a subset of the flock is harvested for slaughter and processing days or weeks before the remainder of the flock. The procedure maximizes stocking density, thereby increasing economic yield, and allows the provision of different-sized chickens to suit market requirements. Two to four cuts per flock are common practice in New Zealand. The close genetic match, high *Campylobacter* prevalence in catching samples, and the timing of flock infection occurring closely following the first cut with catcher presence in the shed all support that catchers and their equipment might have cross-contaminated the raising shed and flock from prior flocks that they visited. There were six STs among the catcher *C. jejuni* isolates sequenced, and both *C. jejuni* and *C. coli* were isolated from catcher samples. While *C. coli* and some STs present in catcher samples were not isolated from the flocks, the catching crew and equipment nonetheless have the potential to introduce new strains to farms and flocks. The results are consistent with other studies and reviews highlighting that thinning is a major risk factor for *Campylobacter* ingress into sheds because it breaches the biosecurity barrier with the catching crews collecting chickens using catching equipment (e.g., cages, modules, trucks, and forklift) and clothes that have been at other farms and/or at the slaughterhouse (7, 21, 22, 49–51). Equipment that enters the shed during catching may also introduce *Campylobacter* from the external farm environment, such as on the wheels of forklifts. The practice of thinning can cause stress for both the captured and remaining chickens, although there are procedures designed to minimize chicken stress set out in the New Zealand Ministry for Primary Industries’ Code of Welfare: Meat Chickens (52). Elevation of the stress hormone noradrenaline in the gastrointestinal tract may promote the growth, proliferation, invasion, and virulence of *Campylobacter* (53). The effect of thinning-induced stress may also explain results from another study. Although enhanced biosecurity practices reduced *Campylobacter* colonization of flocks after the first cut, carcasses from flocks that were thinned were twice as likely to have *Campylobacter* than those that were not (21).

Due to the economic drivers associated with thinning, eliminating the practice of thinning is not seen as a viable option for reducing *Campylobacter* colonization of flocks in New Zealand. Current industry practices require catching crews to change into clean clothes (including hats, beard covers, footwear, and gloves) prior to each site visit (54). In this study, although boots were cleaned and sanitized (via boot dip) between farms, it was noted that the sanitizer required 15 minutes of contact time for maximum efficacy, which was not typically observed, and *Campylobacter* was detected from most boot swabs. All equipment used during harvesting is required to be cleaned to a visibly clean standard (no fecal material, litter, or feathers) and sanitized (completely wetted with sanitizer) between sites, crates should also be dried where practicable, and vehicle interiors should be kept clean. However, in this study, there was a high prevalence of *Campylobacter* from equipment swabs, including from swabs of fecal material visible on crates. Therefore, mitigation options may include improving or ensuring adherence to biosecurity practices during thinning.

Wild and livestock animals proximal to broiler sheds are also a recognized risk factor for *Campylobacter* colonization of chicken flocks (7, 23–25, 55). Although *Campylobacter* isolated from rabbit and wild bird feces proximal to the raising shed did not match isolates from the chicken flocks, there was potential for these to be transmitted into the shed (as discussed above). The role of rodents was not tested because none were trapped over the duration of the experiment, and no rodent droppings were detected around traps, which suggests that rodent control was effective on the farm. Other studies have also reported that crawling and flying insects that commonly frequent broiler sheds, such as darkling beetles and flies, may act as vectors for *Campylobacter* ingress into broiler sheds (26, 56–64). Peak fly presence in summer has been hypothesized to play a role in the seasonal peak of *Campylobacter* prevalence in flocks (61, 65, 66). No flypaper samples tested positive despite pilot experiments determining this to be a sensitive sample type. However, the overwhelming majority of insects captured on flypaper were midges, with fewer small flies and moths, and flies were infrequently observed from sheds. Although both *C. jejuni* ST50 and ST6964 isolates matching isolates from the flock were detected from darkling beetles and larvae, this only occurred following flock colonization when other shed samples also tested positive; thus, it is likely that the flock contaminated the insects, rather than the converse. However, if *Campylobacter* were to enter the shed via just a few darkling beetles, it would be extremely challenging to detect this event because the Arends tubes only collect a small subset of beetles that would be present. Darkling beetles have been reported to persist in sheds following cleaning and disinfection (58, 67). Therefore, the detection of *Campylobacter* in darkling beetles is relevant because they have the potential to transmit *Campylobacter* to subsequent flocks or between sheds.

No evidence was found for vertical transmission of *Campylobacter* in this study. This was based on the absence of *Campylobacter* detection from samples taken from the rearing farm for breeder birds, from the breeder birds 2 weeks post-entry to the breeder facility, or from the chick papers and chick transport equipment during placement in the rearing shed. Although *C. lari* was detected from the breeder birds at the time that the eggs were laid for the study flock, this species was not detected from broiler flocks. *Campylobacter*-contaminated feces from the breeder flocks could contaminate freshly laid fertile eggs, and evidence has been reported that *Campylobacter* can enter egg contents through both transovarian and trans-shell mechanisms, which could be a source of contamination of newly hatched chicks (68). However, *Campylobacter* is isolated very infrequently from chick meconium, fluff, papers, hatchery samples, or from birds younger than 2–3 weeks of age, as discussed elsewhere (69–71). Additionally, sequence types from broiler flocks do not typically match those from their parent breeder flocks, and recent assessments still consider that there remains little to no strong evidence to support egg-borne transmission of *Campylobacter* from the breeder flock (7, 43, 72).

Of the sequence types identified, the most abundant one detected in the study was *C. jejuni* ST6964 (105 isolates), which was found in isolates from the previous, current, and control flocks, as well as catching samples and shed samples once the flock had been colonized. ST6964 was also the predominant ST obtained from carcass rinsates following primary processing and thus has the potential to be present on poultry meat at retail and be relevant from a public health perspective. *C. jejuni* ST6964 was first identified in New Zealand in May 2014 through sentinel surveillance in two retail poultry carcasses sampled in Palmerston North, Manawatu, New Zealand, and emerged contemporaneously in three poultry companies in the North Island of New Zealand (36). Although uncommon in other countries, this ST has since become one of the most frequently isolated from New Zealand poultry flocks and human cases in recent years (5, 36, 73). In a 2019 New Zealand source attribution study, ST6964 was overwhelmingly the most common *C. jejuni* ST from poultry (44 isolates, followed by 24 each for ST45 and ST48) and the fourth most common from human cases (41 cases compared with 119 cases for the most common, ST45) (5). A study that investigated the transmission dynamics of the *C. jejuni* ST6964 lineage through New Zealand poultry flocks reported that the transportation of feed within the commercial poultry industry, as well as other local contacts between flocks, such as the movements of personnel, may have played a significant role in the spread of this strain (73). Although *Campylobacter* was not detected from feed itself in the current study, Greening et al. (73) noted that transportation of feed may be a proxy for other contact networks that were not captured in their analysis. For example, the farms that share the same feed companies may also share the same catching companies, which may result in similarities between the networks (i.e., the movement of transporting feed vehicles and the movement of catching companies).

In this study, there was a high degree of clonality between most ST6964 isolates, consistent with selection (evolutionary bottleneck) from the diverse population in the previous flock, followed by amplification of a successful/escaped clone in the subsequent flock. A minor lineage with only four isolates containing a recombinant region was also present. Indeed, the isolates fit within two of the three distinct lineages identified in an analysis that included genomes from other ST6964 isolates from outside this study, originating predominantly from New Zealand poultry and clinical sources. Two clades of *C. jejuni* ST6964 that were specific to the different poultry companies and differed by the presence of a plasmid (15AR0984-m) were previously identified (36). The authors hypothesized that the *tetO* gene and a phage were inserted into the chromosome from a plasmid after conjugation, leaving a remnant plasmid that was lost from one clade; isolates with the missing plasmid all arose from the same poultry company involved in this study. Plasmid 15AR0984-m contains an operon, including the gene *traC*, which encodes components of a type IV secretion system belonging to the VirB5 family of proteins, responsible for delivering virulence effectors (proteins or protein-DNA complexes) to eukaryotic cell targets (36, 74). Interestingly, while the small cluster of four isolates from the present analysis was missing the plasmid-associated gene *traC*, the predominant ST6964 cluster of isolates was positive for *traC*, suggesting that they did not evolve from the same lineage as the same parent company reported earlier. Although no antimicrobial testing was performed in the current study, *C. jejuni* ST6964 was previously reported to be fluoroquinolone and tetracycline-resistant. All tetracycline-resistant isolates carried a *tetO* allele, which was also found in all ST6964 isolates from this study. As described previously for ciprofloxacin-resistant ST6964 isolates (36), ST6964 isolates from this study also had the C257T (T86I) mutation in *gyrA*, associated with fluoroquinolone resistance (75).

Of the other common sequence types in this study, *C. jejuni* ST50 was also isolated from chickens and *C. jejuni* ST45 from a range of other sample types, and both are commonly isolated from poultry and clinical cases in New Zealand and worldwide (5, 76–78). There were distinct clusters among both *C. jejuni* ST45 and ST50 isolates. The presence of two distinct clades within ST50 has been previously reported, and New Zealand poultry isolates were identified from both clades in a 2019 source attribution study (5, 76). Phylogenetic analyses have also identified a wide diversity among ST45 isolates from different countries and sources (77).

Taken together, this study identifies key areas where the poultry industry might focus on-farm risk management practices to reduce colonization of broiler flocks by *Campylobacter*. The most important areas of focus include farm transmission routes such as carryover from the previous flock or between current flocks and contamination from chicken catching crews and equipment. While the study was carried out on a single farm, the shed design and operating procedures are common or standard throughout the industry, and farm distribution networks (for example, feed, breeder farm and hatchery, catcher company, primary processing plant) supply multiple farms. As such, our findings will likely be representative of transmission routes on other farms and provide key areas for more directed future focus. However, transmission routes for *Campylobacter* contamination of flocks will also vary to some degree by farm location, seasonality, and housing system. Therefore, analogous longitudinal surveys on different broiler farms might identify additional relevant areas for interventions by the poultry industry, toward the goal of reducing the food safety risk for poultry consumers.

## Data Availability

The raw sequence reads were deposited in the National Center for Biotechnology Information (NCBI) archive under BioProject accession number PRJNA1237373 (https://www.ncbi.nlm.nih.gov/bioproject/?term=PRJNA1237373) with the BioSample accession numbers SAMN47418739-SAMN47418936. Sequence assemblies have also been deposited with the accessions JBPDXY000000000-JBPEFM000000000.

## SUPPLEMENTAL MATERIAL

Supplemental material aem.01206-25-s0001.docx Tables S1 to S8, Fig. S1 to S7, and supplemental methods.

ASM does not own the copyrights to Supplemental Material that may be linked to, or accessed through, an article. The authors have granted ASM a non-exclusive, world-wide license to publish the Supplemental Material files. Please contact the corresponding author directly for reuse.

## References

[1] Horn B, Pattis I, Cressey P, Armstrong B, Lopez L. 2023. Annual report concerning foodborne disease in New Zealand 2022. Ministry for Primary Industries, Wellington, New Zealand.

[2] Sears A, Baker MG, Wilson N, Marshall J, Muellner P, Campbell DM, Lake RJ, French NP. 2011. Marked campylobacteriosis decline after interventions aimed at poultry, New Zealand. Emerg Infect Dis 17:1007–1015. doi:10.3201/eid/1706.101272 21749761 PMC3358198

[3] Poultry Industry Association of New Zealand. 2023. NZ poultry production statistics. Available from: https://www.pianz.org.nz/news/poultry-production-statistics/. Retrieved 5 Jul 2024.

[4] Ministry for Primary Industries. 2023. Animal Products Notice: National Microbiological Database Programme. Ministry for Primary Industries, Wellington, New Zealand.

[5] Lake RJ, Campbell DM, Hathaway SC, Ashmore E, Cressey PJ, Horn BJ, Pirikahu S, Sherwood JM, Baker MG, Shoemack P, Benschop J, Marshall JC, Midwinter AC, Wilkinson DA, French NP. 2021. Source attributed case-control study of campylobacteriosis in New Zealand. Int J Infect Dis 103:268–277. doi:10.1016/j.ijid.2020.11.167 33221520

[6] New Zealand Food Safety. 2023. Campylobacter Action Plan 2020-2024. Ministry for Primary Industries, Wellington, New Zealand.

[7] Koutsoumanis K, Allende A, Alvarez‐Ordóñez A, Bolton D, Bover‐Cid S, Davies R, De Cesare A, Herman L, Hilbert F, Lindqvist R, Nauta M, Peixe L, Ru G, Simmons M, Skandamis P, Suffredini E, Alter T, Crotta M, Ellis‐Iversen J, Hempen M, Messens W, Chemaly M, EFSA Panel on Biological Hazards (BIOHAZ). 2020. Update and review of control options for Campylobacter in broilers at primary production. EFS2 18. doi:10.2903/j.efsa.2020.6090 PMC7448041 32874298

[8] European Food Safety Authority. 2010. Analysis of the baseline survey on the prevalence of Campylobacter in broiler batches and of Campylobacter and Salmonella on broiler carcasses, in the EU, 2008 - Part B: analysis of factors associated with Campylobacter colonisation of broiler batches and with Campylobacter contamination of broiler carcasses; and investigation of the culture method diagnostic characteristics used to analyse broiler carcass samples. EFSA J 8:1522. doi:10.2903/j.efsa.2010.1522

[9] Newell DG, Fearnley C. 2003. Sources of Campylobacter colonization in broiler chickens. Appl Environ Microbiol 69:4343–4351. doi:10.1128/AEM.69.8.4343-4351.2003 12902214 PMC169125

[10] Hansson I, Pudas N, Harbom B, Engvall EO. 2010. Within-flock variations of Campylobacter loads in caeca and on carcasses from broilers. Int J Food Microbiol 141:51–55. doi:10.1016/j.ijfoodmicro.2010.04.019 20493571

[11] Grant IH, Richardson NJ, Bokkenheuser VD. 1980. Broiler chickens as potential source of Campylobacter infections in humans. J Clin Microbiol 11:508–510. doi:10.1128/jcm.11.5.508-510.1980 7381018 PMC273443

[12] Stern NJ, Robach MC. 2003. Enumeration of Campylobacter spp. in broiler feces and in corresponding processed carcasses. J Food Prot 66:1557–1563. doi:10.4315/0362-028x-66.9.1557 14503705

[13] Lassen B, Helwigh B, Kahl Petersen C, Ellis-Iversen J. 2022. Systematic review of products with potential application for use in the control of Campylobacter spp. in organic and free-range broilers. Acta Vet Scand 64:24. doi:10.1186/s13028-022-00644-z 36076217 PMC9461118

[14] Soro AB, Whyte P, Bolton DJ, Tiwari BK. 2020. Strategies and novel technologies to control Campylobacter in the poultry chain: a review. Compr Rev Food Sci Food Saf 19:1353–1377. doi:10.1111/1541-4337.12544 33337085

[15] Chinivasagam HN, Estella W, Maddock L, Mayer DG, Weyand C, Connerton PL, Connerton IF. 2020. Bacteriophages to control Campylobacter in commercially farmed broiler chickens, in Australia. Front Microbiol 11:632. doi:10.3389/fmicb.2020.00632 32395115 PMC7197261

[16] Kingsbury JM, Horn B, Armstrong B, Midwinter A, Biggs P, Callander M, Mulqueen K, Brooks M, van der Logt P, Biggs R. 2023. The impact of primary and secondary processing steps on Campylobacter concentrations on chicken carcasses and portions. Food Microbiol 110:104168. doi:10.1016/j.fm.2022.104168 36462824

[17] Lake R. 2006. Transmission routes for campylobacteriosis in New Zealand. A report for the New Zealand Food Safety Authority. Client Report FW0424. Institute of Environmental Science and Research: Christchurch Science Centre.

[18] Agunos A, Waddell L, Léger D, Taboada E. 2014. A systematic review characterizing on-farm sources of Campylobacter spp. for broiler chickens. PLoS One 9:e104905. doi:10.1371/journal.pone.0104905 25171228 PMC4149356

[19] Robyn J, Rasschaert G, Pasmans F, Heyndrickx M. 2015. Thermotolerant Campylobacter during broiler rearing: risk factors and intervention. Compr Rev Food Sci Food Saf 14:81–105. doi:10.1111/1541-4337.12124 33401809

[20] Natsos G, Mouttotou NK, Magiorkinis E, Ioannidis A, Rodi-Burriel A, Chatzipanagiotou S, Koutoulis KC. 2020. Prevalence of and risk factors for Campylobacter spp. colonization of broiler chicken flocks in Greece. Foodborne Pathog Dis 17:679–686. doi:10.1089/fpd.2020.2795 32808818

[21] Georgiev M, Beauvais W, Guitian J. 2017. Effect of enhanced biosecurity and selected on-farm factors on Campylobacter colonization of chicken broilers. Epidemiol Infect 145:553–567. doi:10.1017/S095026881600251X 27873564 PMC9507656

[22] Lawes JR, Vidal A, Clifton-Hadley FA, Sayers R, Rodgers J, Snow L, Evans SJ, Powell LF. 2012. Investigation of prevalence and risk factors for Campylobacter in broiler flocks at slaughter: results from a UK survey. Epidemiol Infect 140:1725–1737. doi:10.1017/S0950268812000982 22631874

[23] Lyngstad TM, Jonsson ME, Hofshagen M, Heier BT. 2008. Risk factors associated with the presence of Campylobacter species in Norwegian broiler flocks. Poult Sci 87:1987–1994. doi:10.3382/ps.2008-00132 18809860

[24] Ellis-Iversen J, Ridley A, Morris V, Sowa A, Harris J, Atterbury R, Sparks N, Allen V. 2012. Persistent environmental reservoirs on farms as risk factors for Campylobacter in commercial poultry. Epidemiol Infect 140:916–924. doi:10.1017/S095026881100118X 21781366

[25] Battersby T, Whyte P, Bolton DJ. 2016. The pattern of Campylobacter contamination on broiler farms; external and internal sources. J Appl Microbiol 120:1108–1118. doi:10.1111/jam.13066 26788933

[26] Refrégier-Petton J, Rose N, Denis M, Salvat G. 2001. Risk factors for Campylobacter spp. contamination in French broiler-chicken flocks at the end of the rearing period. Prev Vet Med 50:89–100. doi:10.1016/S0167-5877(01)00220-3 11448497

[27] Food and Agriculture Organization of the United Nations, World Health Organization. 2024. Measures for the control of Campylobacter spp. in chicken meat - Meeting report. Vol. 46. . Microbiological Risk Assessment Series

[28] Nisa S, Bercker C, Midwinter AC, Bruce I, Graham CF, Venter P, Bell A, French NP, Benschop J, Bailey KM, Wilkinson DA. 2019. Combining MALDI-TOF and genomics in the study of methicillin resistant and multidrug resistant Staphylococcus pseudintermedius in New Zealand. Sci Rep 9:1271. doi:10.1038/s41598-018-37503-9 30718644 PMC6361924

[29] Seeman T, Goncalves da Silva A, Bulach DM, Schultz MB, Kwong JC, Howden BP. Nullarbor Github. Accessed 29 September 2022. https://github.com/tseemann/nullarbor.

[30] Bolger AM, Lohse M, Usadel B. 2014. Trimmomatic: a flexible trimmer for Illumina sequence data. Bioinformatics 30:2114–2120. doi:10.1093/bioinformatics/btu170 24695404 PMC4103590

[31] Souvorov A, Agarwala R, Lipman DJ. 2018. SKESA: strategic k-mer extension for scrupulous assemblies. Genome Biol 19:153. doi:10.1186/s13059-018-1540-z 30286803 PMC6172800

[32] Zhang J, Xiong Y, Rogers L, Carter GP, French N. 2018. Genome-by-genome approach for fast bacterial genealogical relationship evaluation. Bioinformatics 34:3025–3027. doi:10.1093/bioinformatics/bty195 29608746

[33] Letunic I, Bork P. 2021. Interactive Tree Of Life (iTOL) v5: an online tool for phylogenetic tree display and annotation. Nucleic Acids Res 49:W293–W296. doi:10.1093/nar/gkab301 33885785 PMC8265157

[34] Ciccarelli FD, Doerks T, von Mering C, Creevey CJ, Snel B, Bork P. 2006. Toward automatic reconstruction of a highly resolved tree of life. Science 311:1283–1287. doi:10.1126/science.1123061 16513982

[35] Parker CT, Quiñones B, Miller WG, Horn ST, Mandrell RE. 2006. Comparative genomic analysis of Campylobacter jejuni strains reveals diversity due to genomic elements similar to those present in C. jejuni strain RM1221. J Clin Microbiol 44:4125–4135. doi:10.1128/JCM.01231-06 16943349 PMC1698300

[36] French NP, Zhang J, Carter GP, Midwinter AC, Biggs PJ, Dyet K, Gilpin BJ, Ingle DJ, Mulqueen K, Rogers LE, Wilkinson DA, Greening SS, Muellner P, Fayaz A, Williamson DA. 2019. Genomic analysis of fluoroquinolone- and tetracycline-resistant Campylobacter jejuni sequence type 6964 in humans and poultry, New Zealand, 2014-2016. Emerg Infect Dis 25:2226–2234. doi:10.3201/eid2512.190267 31742539 PMC6874264

[37] Bortolaia V, Kaas RS, Ruppe E, Roberts MC, Schwarz S, Cattoir V, Philippon A, Allesoe RL, Rebelo AR, Florensa AF, et al.. 2020. ResFinder 4.0 for predictions of phenotypes from genotypes. J Antimicrob Chemother 75:3491–3500. doi:10.1093/jac/dkaa345 32780112 PMC7662176

[38] Page AJ, Cummins CA, Hunt M, Wong VK, Reuter S, Holden MTG, Fookes M, Falush D, Keane JA, Parkhill J. 2015. Roary: rapid large-scale prokaryote pan genome analysis. Bioinformatics 31:3691–3693. doi:10.1093/bioinformatics/btv421 26198102 PMC4817141

[39] Ferrés I, Iraola G. 2021. Protocol for post-processing of bacterial pangenome data using Pagoo pipeline. STAR Protoc 2:100802. doi:10.1016/j.xpro.2021.100802 34632414 PMC8487088

[40] Schliep KP. 2011. phangorn: phylogenetic analysis in R. Bioinformatics 27:592–593. doi:10.1093/bioinformatics/btq706 21169378 PMC3035803

[41] Tonkin-Hill G, Lees JA, Bentley SD, Frost SDW, Corander J. 2018. RhierBAPS: an R implementation of the population clustering algorithm hierBAPS. Wellcome Open Res 3:93. doi:10.12688/wellcomeopenres.14694.1 30345380 PMC6178908

[42] Castañeda-Gulla K, Sattlegger E, Mutukumira AN. 2020. Persistent contamination of Salmonella, Campylobacter, Escherichia coli, and Staphylococcus aureus at a broiler farm in New Zealand. Can J Microbiol 66:171–185. doi:10.1139/cjm-2019-0280 31721603

[43] Haems K, Strubbe D, Van Rysselberghe N, Rasschaert G, Martel A, Pasmans F, Garmyn A. 2024. Role of maternal antibodies in the protection of broiler chicks against Campylobacter colonization in the first weeks of life. Animals (Basel) 14:1291. doi:10.3390/ani14091291 38731295 PMC11083098

[44] Evans SJ, Sayers AR. 2000. A longitudinal study of Campylobacter infection of broiler flocks in Great Britain. Prev Vet Med 46:209–223. doi:10.1016/s0167-5877(00)00143-4 10913805

[45] Jacobs-Reitsma WF, van de Giessen AW, Bolder NM, Mulder RW. 1995. Epidemiology of Campylobacter spp. at two Dutch broiler farms. Epidemiol Infect 114:413–421. doi:10.1017/s0950268800052122 7781729 PMC2271305

[46] Sahin O, Luo N, Huang S, Zhang Q. 2003. Effect of Campylobacter-specific maternal antibodies on Campylobacter jejuni colonization in young chickens. Appl Environ Microbiol 69:5372–5379. doi:10.1128/AEM.69.9.5372-5379.2003 12957925 PMC194908

[47] Cawthraw SA, Newell DG. 2010. Investigation of the presence and protective effects of maternal antibodies against Campylobacter jejuni in chickens. Avian Dis 54:86–93. doi:10.1637/9004-072709-Reg.1 20408404

[48] Colles FM, Hedges SJ, Dixon R, Preston SG, Thornhill P, Barfod KK, Gebhardt-Henrich SG, Créach P, Maiden MCJ, Dawkins MS, Smith AL. 2021. Parallel sequencing reveals Campylobacter spp. in commercial meat chickens less than 8 days old. Appl Environ Microbiol 87:e0106021. doi:10.1128/AEM.01060-21 34550767 PMC8579978

[49] Sibanda N, McKenna A, Richmond A, Ricke SC, Callaway T, Stratakos AC, Gundogdu O, Corcionivoschi N. 2018. A review of the effect of management practices on Campylobacter prevalence in poultry farms. Front Microbiol 9:2002. doi:10.3389/fmicb.2018.02002 30197638 PMC6117471

[50] Emanowicz M, Meade J, Bolton D, Golden O, Gutierrez M, Byrne W, Egan J, Lynch H, O’Connor L, Coffey A, Lucey B, Whyte P. 2021. The impact of key processing stages and flock variables on the prevalence and levels of Campylobacter on broiler carcasses. Food Microbiol 95:103688. doi:10.1016/j.fm.2020.103688 33397618

[51] Millman C, Christley R, Rigby D, Dennis D, O’Brien SJ, Williams N. 2017. “Catch 22”: biosecurity awareness, interpretation and practice amongst poultry catchers. Prev Vet Med 141:22–32. doi:10.1016/j.prevetmed.2017.04.002 28532990 PMC5450931

[52] 2018. Code of welfare. Meat chickens. Ministry for Primary Industries, Wellington, New Zealand.

[53] Aroori SV, Cogan TA, Humphrey TJ. 2014. Effect of noradrenaline on the virulence properties of Campylobacter species. Int J Microbiol 2014:279075. doi:10.1155/2014/279075 24669220 PMC3942081

[54] Poultry Industry Association of New Zealand. 2015. Biosecurity manual for NZ chicken growers. Poultry Industry Association of New Zealand.

[55] Taha-Abdelaziz K, Singh M, Sharif S, Sharma S, Kulkarni RR, Alizadeh M, Yitbarek A, Helmy YA. 2023. Intervention strategies to control Campylobacter at different stages of the food chain. Microorganisms 11:113. doi:10.3390/microorganisms11010113 36677405 PMC9866650

[56] Barua S, Bailey M, Zhong K, Iduu N, Dormitorio T, Macklin K, Bourassa D, Price S, Hauck R, Krehling J, Kitchens S, Kyriakis C, Buhr RJ, Wang C. 2023. Research note: Role of darkling beetles (Alphitobius diaperinus) and litter in spreading and maintaining Salmonella Enteritidis and Campylobacter jejuni in chicken flocks. Poult Sci 102:103061. doi:10.1016/j.psj.2023.103061 37717478 PMC10514072

[57] Bates C, Hiett KL, Stern NJ. 2004. Relationship of Campylobacter isolated from poultry and from darkling beetles in New Zealand. Avian Dis 48:138–147. doi:10.1637/7082 15077807

[58] Hazeleger WC, Bolder NM, Beumer RR, Jacobs-Reitsma WF. 2008. Darkling beetles (Alphitobius diaperinus) and their larvae as potential vectors for the transfer of Campylobacter jejuni and Salmonella enterica serovar Paratyphi B variant Java between successive broiler flocks. Appl Environ Microbiol 74:6887–6891. doi:10.1128/AEM.00451-08 18791034 PMC2583492

[59] Royden A, Wedley A, Merga JY, Rushton S, Hald B, Humphrey T, Williams NJ. 2016. A role for flies (Diptera) in the transmission of Campylobacter to broilers? Epidemiol Infect 144:3326–3334. doi:10.1017/S0950268816001539 27523647 PMC5080666

[60] Hald B, Sommer HM, Skovgård H. 2007. Use of fly screens to reduce Campylobacter spp. introduction in broiler houses. Emerg Infect Dis 13:1951–1953. doi:10.3201/eid1312.070488 18258057 PMC2876755

[61] Ekdahl K, Normann B, Andersson Y. 2005. Could flies explain the elusive epidemiology of campylobacteriosis? BMC Infect Dis 5:11. doi:10.1186/1471-2334-5-11 15752427 PMC555947

[62] Shane SM, Montrose MS, Harrington KS. 1985. Transmission of Campylobacter jejuni by the housefly (Musca domestica). Avian Dis 29:384–391. doi:10.2307/1590499 4026732

[63] Hald B, Skovgård H, Bang DD, Pedersen K, Dybdahl J, Jespersen JB, Madsen M. 2004. Flies and Campylobacter infection of broiler flocks. Emerg Infect Dis 10:1490–1492. doi:10.3201/eid1008.040129 15496257 PMC3320412

[64] Dale EL, Nolan SP, Berghaus RD, Hofacre CL. 2015. On farm prevention of Campylobacter and Salmonella: lessons learned from basic biosecurity interventions. J Appl Poult Res 24:222–232. doi:10.3382/japr/pfv016

[65] Nichols GL. 2005. Fly transmission of Campylobacter. Emerg Infect Dis 11:361–364. doi:10.3201/eid1103.040460 15757548 PMC3298251

[66] Bahrndorff S, Rangstrup-Christensen L, Nordentoft S, Hald B. 2013. Foodborne disease prevention and broiler chickens with reduced Campylobacter infection. Emerg Infect Dis 19:425–430. doi:10.3201/eid1903.111593 23628089 PMC3647641

[67] Kaufman PE, Burgess M, Rutz DA, Glenister C. 2002. Population dynamics of manure inhabiting arthropods under an integrated pest management (IPM) program in New York poultry facilities—3 case studies. J Appl Poult Res 11:90–103. doi:10.1093/japr/11.1.90

[68] Cox NA, Richardson LJ, Maurer JJ, Berrang ME, Fedorka-Cray PJ, Buhr RJ, Byrd JA, Lee MD, Hofacre CL, O’Kane PM, Lammerding AM, Clark AG, Thayer SG, Doyle MP. 2012. Evidence for horizontal and vertical transmission in Campylobacter passage from hen to her progeny. J Food Prot 75:1896–1902. doi:10.4315/0362-028.JFP-11-322 23043845

[69] Byrd J, Bailey RH, Wills R, Nisbet D. 2007. Recovery of Campylobacter from commercial broiler hatchery trayliners. Poult Sci 86:26–29. doi:10.1093/ps/86.1.26 17179411

[70] Fonseca BB, Soncini RA, Vieira FL, Siqueira MS, Guimarães AR, Beletti ME, Rossi DA. 2006. Campylobacter sp in organs and meconium of day-old broiler chicks derived from naturally infected breeder hens. Rev Bras Cienc Avic 8:265–268. doi:10.1590/S1516-635X2006000400010

[71] Inglis GD, Ramezani N, Taboada EN, Boras VF, Uwiera RRE. 2021. Analysis of Campylobacter jejuni subtype distribution in the chicken broiler production continuum: a longitudinal examination to identify primary contamination points. Appl Environ Microbiol 87:e02001-20. doi:10.1128/AEM.02001-20 33158900 PMC7848917

[72] Callicott KA, Friethriksdóttir V, Reiersen J, Lowman R, Bisaillon J-R, Gunnarsson E, Berndtson E, Hiett KL, Needleman DS, Stern NJ. 2006. Lack of evidence for vertical transmission of Campylobacter spp. in chickens. Appl Environ Microbiol 72:5794–5798. doi:10.1128/AEM.02991-05 16957196 PMC1563688

[73] Greening SS, Zhang J, Midwinter AC, Wilkinson DA, Fayaz A, Williamson DA, Anderson MJ, Gates MC, French NP. 2021. Transmission dynamics of an antimicrobial resistant Campylobacter jejuni lineage in New Zealand’s commercial poultry network. Epidemics 37:100521. doi:10.1016/j.epidem.2021.100521 34775297

[74] Yeo H-J, Yuan Q, Beck MR, Baron C, Waksman G. 2003. Structural and functional characterization of the VirB5 protein from the type IV secretion system encoded by the conjugative plasmid pKM101. Proc Natl Acad Sci USA 100:15947–15952. doi:10.1073/pnas.2535211100 14673074 PMC307673

[75] Wang Y, Huang WM, Taylor DE. 1993. Cloning and nucleotide sequence of the Campylobacter jejuni gyrA gene and characterization of quinolone resistance mutations. Antimicrob Agents Chemother 37:457–463. doi:10.1128/AAC.37.3.457 8384814 PMC187693

[76] Wallace RL, Cribb DM, Bulach DM, Ingle DJ, Joensen KG, Nielsen EM, Leekitcharoenphon P, Stingl K, Kirk MD. 2021. Campylobacter jejuni ST50, a pathogen of global importance: a comparative genomic analysis of isolates from Australia, Europe and North America. Zoonoses Public Health 68:638–649. doi:10.1111/zph.12853 34041858

[77] Bloomfield SJ, Midwinter AC, Biggs PJ, French NP, Marshall JC, Hayman DTS, Carter PE, Mather AE, Fayaz A, Thornley C, Kelly DJ, Benschop J. 2021. Genomic adaptations of Campylobacter jejuni to long-term human colonization. Gut Pathog 13:72. doi:10.1186/s13099-021-00469-7 34893079 PMC8665580

[78] Sopwith W, Birtles A, Matthews M, Fox A, Gee S, Painter M, Regan M, Syed Q, Bolton E. 2008. Identification of potential environmentally adapted Campylobacter jejuni strain, United Kingdom. Emerg Infect Dis 14:1769–1773. doi:10.3201/eid1411.071678 18976567 PMC2630731

