# Supplementary material for: Longitudinal survey investigating vectors and reservoirs for Campylobacter colonization of chickens on a New Zealand broiler poultry farm
Source: Appl Environ Microbiol. 2025 Aug 28;91(9):e01206-25. doi: 10.1128/aem.01206-25 (PMC12442411; doi:10.1128/aem.01206-25)
Supplement: Supplemental material — Tables S1 to S8, Fig. S1 to S7, and supplemental methods. [file aem.01206-25-s0001.docx]

Supplementary information for **Longitudinal survey investigating vectors and reservoirs for *Campylobacter* colonization of chickens on a New Zealand broiler poultry farm.**

Joanne M. Kingsbury, Nigel French, Anne Midwinter, Rebecca Lucas, Maree Callander, Cody P. Hird, Samantha Smith, Kerry Mulqueen, Roy Biggs and Patrick J. Biggs

**SUPPLEMENTARY TABLES**

**Supplementary Table 1. Overview of captured farm variables**

| **#** | **Question/Observation** | **Variable** | **Measures** | **Information source** |  |
| --- | --- | --- | --- | --- | --- |
| **Seasonality^^[[1]](#footnote-2)^^** | | | | | |
| 1 | What is the average external temperature and rainfall over the period of the flock lifespan in the area surveyed? | Rainfall | Daily value: Daily rainfall (mm) | Nearest rainfall gauges? | Roof of shed 2 |
| 2 |  | External temperature | Daily value: Average, maximum and minimum temperature over each 24 hour period | Source of data: nearest gauges?  Do farms report max and min daily external temperatures or temperatures at intervals? | Roof of shed 2 |
| **Farm-level variables** | | | | | |
| 3 | How many other houses are on the farm? | Flock houses | Single value: Number of houses on the farm | Farm manager | 8, 9 in total |
| 4 | What types of domesticated animals are also present on the farm? Where are these animals located relative to the house? | On-farm pets | Single value, unless there is an important change (e.g. a pet entering or leaving the farm): Number of each type of pet animal present on the farm.  (record whether these animals are allowed to roam near the house, and whether regular workers are responsible for their care/have regular contact). | Farm manager, observations | None |
| 5 |  | On-farm livestock | Single value, unless there is an important change (e.g. a herd entering or leaving the farm): For each type of livestock present on the farm (including poultry kept outside the poultry house, e.g. for eggs), record: The type of livestock, the number of each type of livestock, and whether the herd includes young animals (e.g. calves). Estimate the distance between the animals and the house (m). Record which workers are responsible for their care/have regular contact. | Farm manager, observations | None |
| 6 | What types of wild animals are also present on the farm? Where are these animals concentrated relative to the house? | On-farm wild birds | Single value: The types of birds observed on the farm (record any observations of frequented areas, e.g. roosting trees, feeding areas, ponds), any overhanging branches etc. around the house | Farm manager, observations | Rabbits  Birds |
| 7 |  | On-farm rodents | Weekly value: The number and types of rodents, rodent faeces and rodent traps; numbers of rodents killed per week. | Farm manager, observations | Rodent bait boxes – 5 per shed  Occasional sightings |
| 8 | Is there any obvious surface water present on or near the farm? | Surface water - temporary | Daily value: The presence of puddles of standing water and their distance from house (m). | Farm manager, observations | After rainfall – adjacent to the house |
| 9 |  | Surface water - permanent | Single value: The presence of ditches/drains or other surface water (e.g. ponds) and their distance from house (m)  Daily value: Whether ditches/drains hold visible water. | Farm manager, observations | River  c. 50m  Yes the river |
| 10 | What types of animals are on the land bordering the farm? Where are these animals located relative to the house? | Nearby livestock | Single value, unless there is an important change (e.g. a herd entering or leaving the adjacent land): For each type of livestock present on land adjacent to the farm (including any poultry), record: The type of livestock, the number of each type of livestock, and whether the herd includes young animals (e.g. calves). Estimate the distance between the animals and the house (m). | Farm manager, observations | 100m from farm. Dairy cows, no calves |
| 11 | Are any of the neighbouring farms also poultry farms? | Nearby poultry | Single value: Do any of the neighbouring farms raise poultry (for any purpose)? Record yes/no, and what type of poultry (broiler, layer, free range, etc.). Neighbouring farm = the entire farm that shares at least one boundary with the poultry farm. | Farm manager, observations | No  Nearest poultry is a rearing farm c.3km |
| 12 | Are there efforts to separate “dirty” activities such as movement on and off the farm by vehicles, and managing used litter or dead birds? | Activity separation | Single value: Are there designated “dirty” and “clean” areas on the farm, which attempt to separate activities such as vehicle movement, and storage or movement of carcasses/used litter from the house, from activities such as movement or storage of feed and clean litter? | Farm manager, observations | Yes – dead birds |
| 13 | Is the spare litter enclosed? Are there rodent or fly traps present? | Litter store | Single value: How is litter stored on the farm? (fully enclosed shed, shed with large gaps, roof only, walls only, no enclosure....); if litter is not stored on-farm a description of how delivered. | Farm manager, observations | Yes – fully enclosed room for make up for the sheds  Litter delivered on a dedicated truck |
| 14 | Where is used litter taken? | Used litter | Single value: How is litter disposed of after it is cleared from a house? | Farm manager, observations | A contractor spreads onto farmland |
| 15 | Where are dead chickens disposed of? | Bird disposal | Single value: How are dead birds disposed of? | Farm manager | Stored in freezer at farm entrance |
| 16 | Where is feed stored? | Feed store | Single value: How is feed stored on the farm? (fully enclosed shed, shed with large gaps, roof only, walls only, no enclosure....); if feed is not stored on-farm a description of how delivered. | Farm manager, observations | Silos for each shed on the farm. No exposed feed. |
| **House-level variables** | | | | | |
| 17 | Date of previous flock depopulation | Previous flock | Single value: The date of final depopulation of the flock previously held in the house | Farm manager | 18/10/2019 |
| 18 | Was previous flock *Campylobacter*-positive? | *Campylobacter* positive | Single value (if known): Was *Campylobacter* detected in the previous flock? | Farm manager | Yes |
| 19 | Date of cleaning of house after previous flock | Cleaning date | Single value: Date on which the house was cleaned after final depopulation of the flock previously held in the house | Farm manager | 19/10/2019 – 28/10/2019 |
| 20 | Cleaning procedures. Type of cleaners/sanitisers/how feeders and drinkers cleaned. | Cleaning | Single value: Description of the type of cleaning products used, how applied, and how cleaning of feeders and drinkers is undertaken. | Farm manager | Water and sanitiser |
| 21 | Number of birds in flock | Flock start size | Single value: Number of birds in flock at day 0 (record date) | Farm manager | 18000 F  19300 M  Total 37300 |
| 22 |  | Flock cut | Single value: Number of birds in flock at day of depopulation, number after depopulation (record date); record for each depopulation event | Farm manager | 05/12/2019: 8135 F  19300 M  11/12/2019 – 0 F  19300 M  16/12 0 birds |
| 23 |  | Flock final size | Single value: Number of birds in flock at day of final depopulation | Farm manager | 18805 |
| 24 | Characteristics of the house | House size | Single value: House floor size (m^2^) | Farm manager | 2192 m^2^ |
| 25 |  | House age | Single value: How old is the house? (could be some major alterations since built) | Farm manager | Built in 2014 |
| 26 |  | House layout | Single value: What areas are in the entire house? (e.g. annexe, other divisions) | Farm manager, observations | Annexe is outside the shed 10m x 4m  Tunnels are 2 x 16m x1m |
| 27 |  | Outside house | Single value: What substrate is outside the house entrance? (e.g. concrete, gravel, grass) | Observations | Personnel pathway - Gravel  End pads concrete |
| 28 | Litter changes over time | Litter start | Single value: When was the new litter placed into the shed? Record source of litter (from on-farm storage or delivered straight to shed) | Farm manager | 31/10/2019  Delivered straight to shed |
| 29 |  | Litter refresh | Daily value: Was there any new litter added to the house? | Farm manager | Yes – frequent *occasions as necessary recorded in farm diary |
| 30 | House vents | Vents | Single value: How many vents are in the house? Brief description: Horizontal or vertical, with or without fans (blow in or out?) | Farm manager, observations | 136 vents, horizontal,  Air is sucked into the shed |
| 31 |  | Vent open | Daily value: How long are the vents open/running? | Farm manager | Variable, as required |
| 32 |  | Vent net | Single value: Are there any nets or similar covering the vents to prevent insects moving into the shed? | Farm manager, observations | No bird screened - plastic |
| 33 | What is the source and treatment of the water? | Water source | Single value: Where does the flock’s drinking water come from? Bore, surface, municipal supply. | Farm manager | River and bore |
| 34 | Characteristics of the drinking water | Water system | Single value: Describe the drinking water system (troughs, lines with nipples, lines with nipples+bells/cups, etc.) | Farm manager, observations | Nipple drinkers |
| 35 |  | Water treatment | Is the water treated? Yes/no. Specify treatment type. | Farm manager | Chlorinated near source and then chlorine dioxide before entry to shed |
| 36 | Feed delivery | Feed system | Single value: Describe the feed delivery system (are there any places where the feed is exposed to the external environment?) | Farm manager, observations | Feed tankers deliver into the feed silos. Enclosed – no exposure |
| 37 | How many workers/visitors entered the house? | Workers | Single value: Number of regular workers who enter the house daily. | Farm manager | Dependent on tasks, usually 4 or 5 |
| 38 |  | Visitors | Daily value: Number of visits by people who are not regular workers and who entered the house.  (record date visit occurred and reasons for visit, e.g. vet, inspection, delivery, thinning crew, temporary worker). | Farm manager | Recorded and provided in the farm diary*  Electrician, Tegel broiler service people |
| 39 | What equipment was brought into the house? | Equipment | Daily value: What equipment/vehicles entered the house? (includes tools, machinery, vehicles, crates)  Is this equipment shared between houses? (yes/no)  If yes, is this equipment sanitised before it is taken into the house? | Farm manager | Scales, bucket  Rotary hoe  Yes  No |
| 40 | Were any birds/flies/beetles/rodents observed it the annexe or shed during each visit? Signs of birds in the shed e.g. droppings? | In-house wild animals | Daily value: Were any wild birds, rodents, or their droppings observed in the house, annexe or in/on any traps? | Farm manager, observations | None, 2 birds caught in rat traps outside shed |
| 41 |  | In-house insects | Daily value: Were any insects observed in the house or in/on any traps? | Farm manager, observations | Results on sample set A reports |
| **Biosecurity measures** | | | | | |
| 42 | Boot wash/boot covers/dedicated boots per shed? | Boots | Single value: What procedures are in place to manage footwear? (boot covers, boot wash, etc.)  If a boot wash is present: How often is the wash cleaned out/refreshed? | Farm manager, observations | Boot exchange every shed |
| 43 | How often are overalls washed/changed? | Clothing | Single value: Are there any requirements to change clothing before entering the house? | Farm manager, observations | Daily or more frequently if soiled |
| 44 | Is there an order of movement of personnel between houses? | Movement | Single value: If multiple houses, is there a set order that workers move between houses? (e.g. starting at house with youngest flock) | Farm manager | No – as required |
| 45 | Are there hand wash stations outside each shed and how are these used? | Hands | Single value: What facilities are in place for hand washing and how are workers required to use these? | Farm manager, observations | Hand wash (soap & water) and hand sanitiser |
| 46 | Are biosecurity procedures consistently applied? | Biosecurity test | Single value: Are visitors asked to adhere to boot, clothing and hand washing procedures? | Observer (researcher) | Yes |
| 47 | What types/how many rodent traps are present in the shed/annexe/vicinity? | Rodent traps | Single value: Are there rodent traps in the house/annexe or nearby? What types? | Farm manager, observations | Vicinity – 5 rodent bait boxes outside all sheds |
| 48 | Are there fly traps in the annexe and/or shed? | Fly traps | Single value: Are there fly traps in the house/annexe or nearby? What types? | Farm manager, observations | No |
| 49 | How often are dead birds removed from the shed? | Dead birds | Daily value: Number of dead birds removed from the house. | Farm manager | Daily |
| **Personnel** | | | | | |
| 50 | Is there a stand down imposed between visits to the farm from people that have been on other poultry farms? If not, what are the requirements for changes to clothes/boots/washing of equipment? | Stand down | Single value: Is there a stand down period imposed between visits to the farm from people that have been in contact with poultry on farms? (yes/no)  NB: This includes workers who also work on other poultry farms or keep their own poultry.  If Yes: Detail.  If No: Are there any additional biosecurity requirements imposed on these people? | Farm manager | Yes 36 hours from breeder farms and non-Tegel farms. No standdown between Tegel broiler farms.  Workers not permitted to keep any avian species |
| 51 | Training of personnel with respect to *Campylobacter* and biosecurity measures? | Training | Single value: Are workers specifically trained to understand *Campylobacter* and how to minimise the chance of a flock being colonised? | Farm manager | Element of training and is part of the Health & Safety system.  Biosecurity training |

**Supplementary Table 2.** Sample types and locations to be tested, testing methodology, target sample numbers and rationale for testing.

| **Sample location** | **Sample type** | **Sample equipment/type/setup** | **Sample collection** | **Sampling times and numbers** | **Rationale for testing** | **Sample set^^[[2]](#footnote-3)^^** | **Target sample numbers** |
| --- | --- | --- | --- | --- | --- | --- | --- |
| Breeder rearing shed (where breeder chickens spend the first ~18-20 weeks prior to coming into lay) | Boot sock pair | Boot sock pairs comprised disposable, elasticized hair covers (Med-X round bouffant, Fabri-cell).^^[[3]](#footnote-4)^^  Boot socks were autoclaved, then pre-moistened with 20 ml Maximum Recovery Diluent (MRD) by the testing laboratory. | After entering area to be sampled, fresh plastic boot covers (Nasco, Hardy Diagnostics, US) placed over boots (to protect sample from boot contamination and boot dip chemicals). Using gloved hands, boot socks placed over plastic boot covers. Designated sample area walked around, up one row (between feeders) to the end wall and walk back another row. With gloved hands, boot sock pair placed back into original sample bag. | 2 samples prior to breeder flock transferral into raising shed. | Potential reservoir |  | 2 |
| Breeder production shed (where breeder chickens housed when laying eggs) | Boot sock pair | Boot socks described above | Method described above | 4 samples of empty shed prior to placement of breeder flock, 4 samples 2 weeks following transfer, 4 samples on date that eggs destined for shed K2 flock are laid. | Potential reservoir |  | 12 |
| Broiler shed K2 | Paper lining from chick transport crates |  | 2 randomly selected pieces of paper collected together with associated droppings, feathers, etc and pooled together into 1 large Whirl-Pak bag. Repeated for 5 samples (i.e. 10 total pieces of lining paper; total surface area should be at least 1 m^2^). Ideally, sample taken before entering broiler raising shed to eliminate chance that cross-contamination occurred from shed to transport crates. If sample is taken in shed, transferred directly to Whirl-Pak bag immediately after chick transfer. | 5 samples on day of transfer. | Potential reservoir | F | 5 |
| Broiler shed K2 | Boot sock pair | Boot socks described above | Method described above | 2 samples from previous flock near depopulation, 2 samples prior to placement of broiler flock after cleaning and application of fresh litter, 2 samples every 5 days after placement of chicks. | Potential reservoir (previous broiler flock and empty shed), evidence of flock colonization (current broiler flock) | A | 22 |
| Broiler shed K2 | Cloacal swabs | Amies transport swab with charcoal (Copan Murrieta, CA, USA). | Chickens selected from close to 12 numbered areas on the wall of the shed. Swabs placed back into respective transport tube and each set of 5 swabs/transport tubes from a single location pooled in a standard Whirl-Pak bag. | 12 pools of 5 swabs (60 swabs total) every 5 days from day 5 to day 40 (i.e. total of 480 chickens sampled^^[[4]](#footnote-5)^^). | Evidence of flock colonization, types colonizing | A | 96 |
| Broiler shed K2 | Water supply | 2 x 1 L bottles with sodium thiosulphate (to neutralize any chlorine present in water). | Prior to water collection, water flushed from both collection sources for ~10 seconds. 1 L collected from the shed source and 1 L flushed from a drinker line. | 2 samples collected following cleaning and sanitation prior to placement of broiler flock, 2 samples every 5 days during broiler raising. | Potential reservoir | A | 18 |
| Broiler shed K2 | Nipple drinkers | 3M^TM^ Enviro-swab. | 12 drinker nipples swabbed from each of 6 drinker lines. Both sides of single swab used for all drinker nipples per line. Swabs returned back to transport tubes. | 5 samples prior to placement of broiler flock, 6 samples every 5 days during broiler raising. | Potential reservoir | A | 53 |
| Broiler shed K2 | Shed floor, vents, fans, surfaces | 3M^TM^ Enviro-swab | At least 20 areas swabbed that were potential harbourage sites, e.g. floor cracks, hard-to-clean spaces, shed corners. Sample location indicated on sample bag/sample sheet. | 5 samples following cleaning and sanitation after shed has dried, prior to placement of litter. | Potential reservoir | D | 20 |
| Broiler shed K2 | Worker clothing^^[[5]](#footnote-6)^^ | 3M^TM^ Enviro-swab, 1 cotton apron. | One farm worker that accessed the broiler sheds (e.g. to check for dead chickens) was provided with a cotton apron. They were asked to wear this over their normal clothes for first two sheds that they enter. On entry to the study shed (K2) the apron was removed and placed in a sterile bag. The front of the apron and any soiled areas was be swabbed (1 or 2 swabs, dependent upon soiling) and the swab(s) returned to the transport tube and sent to the laboratory. | 1 sample every 5 days during broiler raising (either on the day of sampling or the day prior to collection). | Potential vector | A | 8 |
| Broiler shed K2 | Flies/flying insects | Flypaper (Number 8 brand, Mitre 10, New Zealand; poison-free). Hung approx. 5 days before sampling at 6 locations. | Using scissors that have been sterilized using an alcohol swab, ~5-10 cm length of flypaper cut containing the highest number of insects and carefully placed at bottom of Whirl-Pak sampling bag. Remainder of flypaper sample discarded. | 6 samples every 5 days during broiler raising, if insects present on flypaper. | Potential vector | A | 48 |
| Broiler shed K2 | Darkling beetles/crawling insects | Sanitized Arends tube traps consisting of PVC pipe tubes filled with rolled corrugated cardboard, were manufactured at Massey University. 12 tubes placed in different sites within shed near walls, doors and annex, particularly at gaps and sites where insect incursion might occur, approx. 48 hours before sampling. | Ends of tubes capped. Cardboard inner removed from tube and placed into a plastic bag. CO_2_ (from CO_2_ cylinder stored outside broiler shed) injected and bag sealed with a cable tie, to euthanize insects prior to transit to laboratory. | 12 samples every 5 days during broiler raising, if insects present in traps.  Alternatively, contents from 2 traps pooled and always pool from the same 2 sites. | Potential vector | A | 96 |
| Broiler shed K2 annex | Shed entry/annex | 3M^TM^ Enviro-swab | 3 locations of the annex swabbed with individual swabs (i.e. 3 separate samples); sampling area demarcated with tape so that same site sampled each time:   - 1 swab at the entry into annex from outside (swab a zone of ~100 cm^2^), - 1 swab from boot change by entry into broiler shed, (swab a zone of ~100 cm^2^), - 1 swab of multiple areas where workers’ hands will have touched, e.g. door handle, wall area near entry to shed where workers will hold for balance while putting on boots. - 1 swab of annex caddy.   Areas sampled indicated on sample and sample sheet. Each sample returned to transport tube supplied with swab. | At least 3 samples every 5 days during broiler raising. | Evidence of cross-contamination from outside to annex or shed to annex | A | 24 |
| Broiler shed K2 annex, litter storage room, feed weigh room, perimeter | Rodents | 6 rat traps placed around shed perimeter and 2 mouse traps in the annex, 1 mouse trap in feed weigh room, 2 mouse traps in litter storage room. Reset and bait with fresh peanut butter approx. 48 hours before sampling. | Viscera (complete with liver) removed from any trapped rodents. One per large Whirl-Pak bag, labelled with location. | Up to 4 samples every 5 days during broiler raising. | Potential vector | A | 0-32 |
| Surrounds of broiler shed K2 | Wild birds  (found dead outside shed) |  | Assess for dead wild birds surrounding shed. Viscera removed from any wild birds found dead around the shed. One per large Whirl-Pak bag and label. | Every 5 days during broiler raising. | Potential reservoir | A | 0-5 |
| Surrounds of broiler shed K2 | Wild birds (feces) | Large tarpaulin placed on the ground near shed K2 ~48 hours before sampling.  Amies transport swab with charcoal (Copan, Murrieta, CA, USA)^^[[6]](#footnote-7)^^. | Feces present on tarpaulin/paper swabbed using Amies transport swab with charcoal. Up to 5 droppings pooled per sample.  Swab returned to sample tube. Number of individual droppings sampled recorded on in the sample.  If no droppings found on tarpaulin, any fresh wild bird feces observed on or around the shed swabbed with Amies transport swab with charcoal. Swab returned to sample tube and location of sample collection recorded. | At least 1 sample every 5 days, if droppings detected. | Potential reservoir | A | >8 |
| Surrounds of broiler shed K2 | Soil sample using boot sock pair | Boot socks described above | Boot socks placed over plastic boot covers, as described above. For the first sample, wearing boot socks, entire area walked including annexe entrance, weigh room and feed silos. For the second sample, walked around areas around exhaust fans (end pads and the full length of the shed on the opposite side to the annex), stepping on any observed dust exhausted from shed. | 2 samples every 5 days during broiler raising. | Potential reservoir (sample from annex vicinity), evidence of egress from shed (fan exhaust area) | A | 16 |
| Broiler shed K2, K3 | Catching crew cloth glove pair (or overalls) from previous farm | Cotton gloves | If catching crew were involved in depopulation at another farm on the same day, gloves requested that were used for capturing chickens from prior farm. Gloves placed in large Whirl-Pak bag, 2 pairs of gloves per bag. Samples were non-identifiable. | 2 samples per cut (K2); K3 only sampled after first cut. | Potential reservoir. Although the same gloves are not used between flocks, informs on what ST were present in previous flock. | B | 4 |
| Broiler shed K2, K3 | Catching crew boot swabs | 3M^TM^ Enviro-swab | External soles of one boot for each of 6 catchers and/or catcher crew swabbed before entry into shed K2, and ideally, before they step out of the transport van. Workers not identified. | 6 samples per cut (K2); K3 only sampled after first cut. | Potential reservoir | B | 24 |
| Broiler shed K2, K3 | Catching truck tyres, wheel arches and ledges, seat and steering wheel | 3M^TM^ Enviro-swab | Upon arrival of truck at shed and prior to catching commences, 2 swab samples collected.   - Swab 1: steering wheel and truck seat - Swab 2: wheels, wheel arches, and truck ledges | 2 samples per cut (K2); K3 only sampled after first cut. | Potential reservoir | B | 8 |
| Broiler shed K2, K3 | Forklift transporting catching crates and modules | 3M^TM^ Enviro-swab | Before the forklift enters the shed, collect 3 sets of swabs from forklift.   - Swab 1: Swab of tyres, dusty ledges, wheel arches and forks - Swab 2: Swab seat and steering wheel | 3 samples per cut (K2); K3 only sampled after first cut. | Potential reservoir | B | 12 |
| Broiler shed K2, K3 | Catching crates and modules | 3M^TM^ Enviro-swab | Before the modules and crates transported into the shed, surfaces swabbed from the floor of 5 crates, 5 samples swabbed of visible fecal material from crates, and 5 modules swabbed. | 15 samples per cut (K2); K3 only sampled after first cut. | Potential reservoir | B | 55 |
| Broiler shed K2, K3 | Cecal contents |  | Two ceca from each of 5 chickens culled for coccidiosis check removed and pooled both ceca per chicken in a large Whirl-Pak bag (i.e. 5 samples per shed, 10 samples for 2 sheds). Alternatively, 10 ceca pooled from all 5 culled chickens per shed (i.e. 1 sample per shed, 2 samples for both sheds). | 10 samples at time of coccidiosis check at 17-22 days of age. | Evidence of colonization, determine whether *Campylobacter* from shed K3 is the same as that from shed K2 |  | 40 |
| Processing plant | Cecal contents |  | 1 ceca removed from each of 10 chickens after evisceration. If the selected ceca looked small, discarded and another sample selected. Each ceca placed into an individual Whirl-Pak bag (i.e. 10 individual samples per shed; 20 for each cut/2 sheds). For chickens arising from previous flock from Shed K2, 10 ceca from 10 chickens collected. | Following slaughter at evisceration, each cut for chickens from both Shed K2 and Shed K3, and previous flock from Shed K2. | Evidence of colonization, and source of colonizing sequence types. | C | 70 |
| Processing plant | Broiler rinsate |  | 15 rinsate samples arising from 15 carcasses from processing of shed K2; 6 rinsate samples arising from 6 carcasses from K3 chickens collected after each cut (sampling methodology as used for National Microbiological Database sampling (1) ). | During processing after each cut. | Evidence of types that survive processing interventions. | C | 63 |
| Feed weigh that supplies K2 | Feed and feed dust |  | Two samples of feed collected from 50-80 mm below the surface from each of 2 feed weigh hoppers using sanitized scoop, and 1 sample of feed dust collected from frame of weigh hoppers. | 3 samples every 5 days.^[[7]](#footnote-8)^ | Potential reservoir. | A |  |
| K2, surrounds | Other | Various | Opportunistic samples collected that might constitute vectors or reservoirs, e.g. swabs of feed trucks entering farm, rabbit feces. | Variable | Other potential reservoirs or vectors. |  |  |

**Supplementary Table 3.** Laboratory processing and testing of each sample type for *Campylobacter.*

| **Sample type** | **Sample processing by laboratory** | **Sample enrichment and testing** |
| --- | --- | --- |
| Boot sock pair |  | 200 ml Bolton broth added to boot sock pairs, stomached for 2 min prior to enrichment. |
| Flies | 10 ml of MRD added to flies and flypaper. Numbers and types of flies present on the flypaper recoded. | Flies on flypaper macerated within MRD buffer in the Whirl-Pak bag using three rolls of a rolling pin. 90 ml volume of Bolton broth added. |
| Beetles and other crawling insects | Arends tubes disassembled and insects transferred using sterile forceps to Whirl-Pak bag containing 10 ml MRD. Up to 10 insects per Arends tube per test unit. | Insects macerated within MRD buffer in Whirl-Pak bag using three rolls of a rolling pin. 90 ml volume of Bolton broth added. |
| Paper lining from chick transport crates |  | Processed on day of sample receipt. 200 ml Bolton broth added to sample and stomached for 2 min prior to enrichment. |
| Drinking water | On the day of enrichment, 1 L water sample filtered using sterile 0.45 µm nitrocellulose filter. | Filter placed into 100 ml Bolton broth and stomached for 2 min prior to enrichment. |
| Wild bird gut packs | Contents weighed. | Bolton broth added in 1:9 ratio (e.g. 9 ml to 1 g sample). Stomached for 2 min prior to enrichment. |
| 3M^TM^ Enviro-swab |  | Enviro-swabs removed from the applicator stick and added to 30 ml pot containing 20 ml Bolton broth. |
| Day-to-day worker disposable overalls | Pair of overalls swabbed using single Enviro-swab per leg (particularly below the knees), arms including cuff area, and front of overalls. | Enviro-swabs removed from the applicator stick and added to 30 ml pot containing 20 ml Bolton broth. Applicator stick container rinsed with a portion of the Bolton broth to recover remaining sample. |
| Catcher gloves | Single Enviro-swab per pooled sample swabbed over outside surfaces of catcher gloves. | Enviro-swabs removed from the applicator stick and added to 30 ml pot containing 20 ml Bolton broth. Applicator stick container rinsed with a portion of the Bolton broth to recover remaining sample. |
| Cloacal swabs |  | 5 swabs per pooled sample combined into single 30 ml pot containing 20 ml Bolton broth. |
| Wild bird feces |  | Swab sample placed into 30 ml pot containing 20 ml Bolton broth. |
| Cecal contents | 1 g cecal contents arising from individual chickens weighed out and mixed thoroughly. | Well-mixed sample material plated onto two halves of a mCCDA agar plate using two sterile loops. Bolton broth (9 ml) added to well-mixed sample. Enrichment broth plated if *Campylobacter* colonies not obtained following direct plating. |
| Broiler rinsate |  | Rinsate plated directly upon receipt of samples, 333 µl per plate over 6 mCCDA plates. 90 ml Bolton broth added to 10 ml rinsate for enrichment. |
| Feed | Weigh 10 g of feed. | 90 ml Bolton broth added to 10 g feed. Stomached for 2 min prior to enrichment. |

**Supplementary Table 4.** Activities occurring in broiler raising shed K2 (or breeder sheds and control K3 shed, where specified) throughout the study period.

| **Flock age (days)** | **Activities in raising shed and flock sampling** |
| --- | --- |
| -120 | Sampling of rearing shed of breeder flock, and breeder flock laying shed of flock pre-placement; boot sock samples |
| -99 | Sampling of breeder flock laying shed two-weeks post flock placement; boot sock samples |
| -25 | Sampling of breeder flock laying shed during egg collection for study broiler flock; boot sock samples |
| -19 | Flock clearance; boot sock samples |
| -18 | Flock clearance; ceca collected at processing plant |
| -7 | K2 shed sampled post-cleaning, sanitation and drying, before addition of litter; sample set D |
| -5 | K2 shed sampled after addition of litter; boot socks, drinker swabs, water samples |
| 0 | Placement of 38,000 chicks |
|  | Sample set F |
|  | Feed truck delivery; steering wheel and foot pad swab |
| 1 | Water leak in male end, litter dug out and 3 bags used |
|  | RH and CO_2_ sensor in the wrong ports |
|  | Feed truck delivery; steering wheel and foot pad swab |
| 2 | Controller glitch with low temperature |
|  | Water leak in female end, nipple replaced, litter dug out and replaced; 2 bags of litter added |
| 3 | Bird nest found in 4^th^ vent male end |
|  | Brooding paper placed into piles |
| 4 | Raked shavings under the drinkers |
|  | Feeders turned off for short period |
|  | Water leak dug out and 1 bag of litter added |
| 5 | Sample set A and extras (e.g. brooding paper). No flying insects on fly paper. |
|  | Lighting changed to 19 on and 5 off |
| 6 | Electrician in shed; ladder in and out |
|  | Leak in male end; ½ bag of litter added |
| 7 | Litter forked, rakes taken in and out of shed |
|  | Chickens weighed; bucket, tablet and hand scales in and out of shed |
|  | Dug out leak; 3 bags of litter added |
| 8 | Leak, dug out; 1 bag of litter added |
| 10 | Sample set A |
|  | 60 bags added to top dress litter |
|  | Litter forked in whole shed |
|  | Leak, dug out; 2 bags of litter added |
| 11 | Forked a small area of shed |
| 13 | Leak, dug out; 1 bag of litter added |
|  | Heater 1 failed and repaired |
|  | Litter delivery truck; steering wheel and foot pad sampled |
| 14 | Raked whole shed, rake in and out of shed |
|  | Chickens weighed; bucket, tablet and hand scales in and out of shed |
| 15 | Sample set A |
|  | No crawling insects observed but the quality of the litter was becoming much more consolidated which may provide greater cover for the darkling beetles. A few more insects observed (predominantly small black midges). The air inlet vents were not fully open yet and the lighting cycles at night expected to capture more moths over time. |
|  | Chickens culled for coccidiosis check; cecal samples tested for K2 and K3 |
|  | Vents fixed; cord taken in and, and socket set taken in and out of shed |
|  | 2 wild birds’ nests taken out of river side vents |
| 17 | 60 bags of shavings used to top dress litter |
| 18 | Outside rows of drinkers lowered |
|  | Litter forked |
|  | Wild birds nests removed from tunnels |
|  | Growing chickens now utilising more of the shed |
| 20 | Sample set A |
|  | Chickens culled for coccidiosis check; cecal samples tested for K2 and K3 |
|  | Increase in insect activity (crawling and flying) |
|  | Rotovated litter, rotavator in and out of shed |
| 21 | Chickens weighed; bucket, tablet and hand scales in and out of shed |
| 22 | Wet litter removed and replaced with 2 bags of dry litter |
| 23 | Repaired barrier netting which separates males from females |
|  | Litter added; 6 wool bags and 10 black sacks |
| 25 | Sample set A and extras |
|  | LPG delivery truck, steering wheel and foot pad sampled |
|  | Wild birds’ nest found |
| 28 | Sample set A |
|  | Boot sock samples; K3 |
|  | Chickens culled for coccidiosis check; cecal samples tested for K2 and K3 |
| 29 | 28 bags shavings added to top dress litter |
|  | K2 first cut; sample sets C (catchers and catching equipment) and D (in processing plant) |
| 30 | Fixed broken rope on vents; tools taken in and out of shed |
|  | 1 wild birds’ nest found |
|  | K3 first cut; sample sets C (catchers and catching equipment) and D (in processing plant) |
| 34 | Boot sock samples; K3 |
| 35 | Chickens culled for coccidiosis check; cecal samples tested for K2 and K3 (males) |
|  | Sample set A and extras |
|  | K3 second cut; sample set D (in processing plant) |
| 36 | K2 second cut; sample sets C (catchers and catching equipment) and D (in processing plant) |
|  | Moffat forklift and cones and barriers remained on-site overnight. |
| 38 | K3 final cut; sample set D (in processing plant) |
| 40 | Chickens weighed; bucket, tablet and hand scales in and out of shed |
|  | Sample set A |
| 41 | K2 final cut; sample sets C (catchers and catching equipment) and D (in processing plant) |

**Supplementary Table 5.** Numbers and types of insects caught in flypapers and Arends tubes at sampling points. All insects captured were sampled unless specified otherwise. All crawling insects were darkling beetles or their larvae; flying insects were described based on appearance but were not described to a taxonomic level.

|  | **Sample** | **Flock age (days)** | | | | | | | |
| --- | --- | --- | --- | --- | --- | --- | --- | --- | --- |
|  |  | **5** | **10** | **15** | **20** | **25** | **28** | **35** | **40** |
| Flying  insects (flypaper) | 1 | 0 | 1 small fly | 4 midges | 37 midges | 1 large midge,  20 small midges,  1 long-legged insect | 28 midges,  1 long-legged fly | 1 moth,  1 large midge,  35 small midges | 31 small midges,  1 moth,  1 large midge,  1 mosquito |
|  | 2 | 0 | 0 | 0 | 41 midges | 39 midges;  5 sampled | 1 large midge,  25 small midges | 24 small midges;  not sampled | 24 small midges,  1 fly |
|  | 3 | 0 | 0 | 5 midges;  2 sampled | 11 midges | 28 midges, 1 moth | 2 large midges,  8 small midges,  not sampled | 9 small midges | 14 small midges,  1 small fly |
|  | 4 | 0 | mosquito | 1 moth,  1 midge | 26 midges | 27 midges;  15 sampled) | 12 small midges,  not sampled | 9 small midges;  not sampled | 4 small midges,  not sampled |
|  | 5 | 0 |  | 0 | 39 midges | 46 midges | 1 beetle,  7 midges | 22 small midges | 14 small midges,  1 moth |
|  | 6 | 0 |  | 3 midges;  2 sampled | 29 midges | 1 moth, 1 fly, 32 midges | 17 midges | 1 fly,  18 small midges | 24 small midges,  3 small flies |
| Crawling insects  (Arends tubes) | 1 | 0 | 0 | 0 | 2 larvae | 1 beetle, 14 larvae | 21 larvae;  10 sampled | 0 | 0 |
|  | 2 | 1 beetle | 0 | 0 | 7 larvae | 3 beetles, 13 larvae | 17 larvae;  10 sampled | 1 beetle, 1 larvae | 0 |
|  | 3 | 1 beetle | 0 | 0 | 9 larvae | 0 | 31 larvae;  10 sampled | 5 larvae | 1 larvae |
|  | 4 | 0 | 0 | 0 | 0 | 0 | 2 larvae | 0 | 0 |
|  | 5 | 0 | 0 | 0 | 0 | 2 beetles, 2 larvae | 0 | 0 | 0 |
|  | 6 | 0 | 0 | 0 | 0 | 0 | 0 | 0 | 0 |
|  | 7 | 0 | 0 | 0 | 2 larvae | 1 beetle, 8 larvae | 11 larvae;  10 sampled | 0 | 0 |
|  | 8 | 0 | 0 | 0 | 0 | 0 | 1 beetle,  12 larvae;  1 beetle and  10 larvae sampled | 0 | 0 |
|  | 9 | 1 beetle | 2 beetles | 0 | 26 larvae;  10 sampled | 6 larvae | 0 | 1 beetle, 4 larvae | 0 |
|  | 10 | 0 | 0 | 0 | 2 larvae | 1 larvae | 3 larvae | 0 | 0 |
|  | 11 | 0 | 1 beetle | 0 | 1 larvae | 1 beetle, 1 larvae | 0 | 0 | 1 beetle, 2 larvae |
|  | 12 | 0 | 0 | 0 | 5 larvae | 0 | 8 larvae | 0 | 0 |

**SUPPLEMENTARY FIGURES**


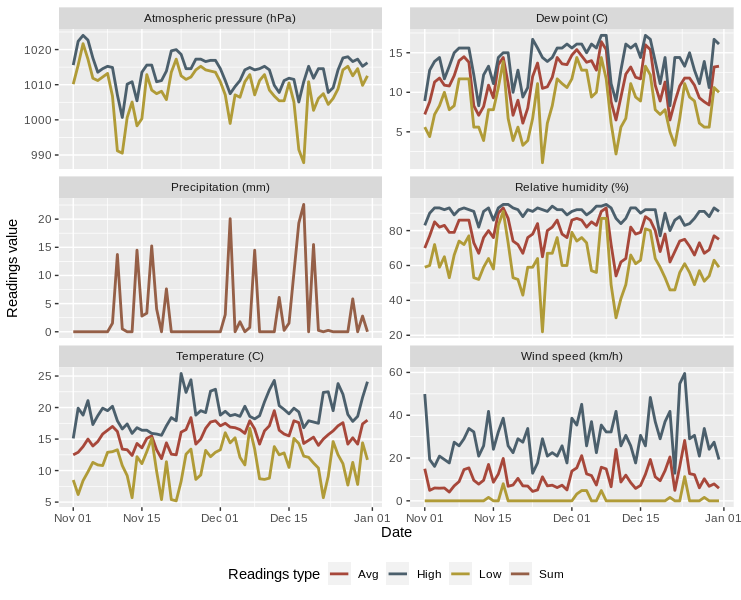


**Supplementary Figure 1.** Weather variables for the closest observation point to the study farm for the period November and December 2019.





**Supplementary Figure 2.** Phylogenetic relationships of *C. jejuni* ST6964 isolates based on Single Nucleotide Polymorphism distance using *C. jejuni* 15AR0984 (ST6964) as the reference genome, with associated sampling time and sample type metadata.


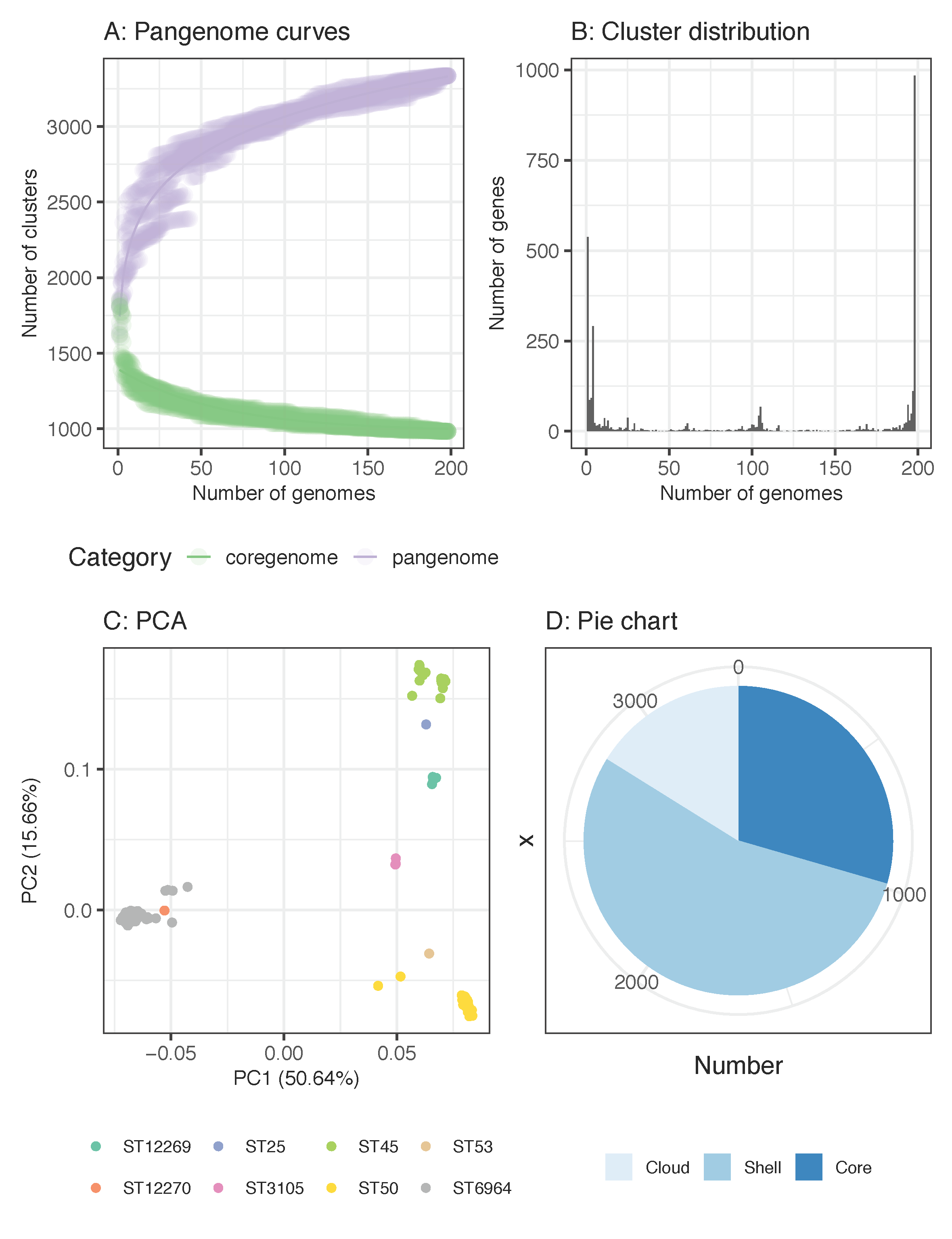


**Supplementary Figure 3.** Summary of the pangenome generated from the 198 isolates in this study. A) Plots of core genome and pangenome for the 198 isolates. B) Barplot with the frequency of genes within the total number of genomes. C) Principal component analyses (PCA) of the isolates coloured by ST. D) Pie chart showing the distribution of the 3336 gene clusters in the pangenome by category: Core – 984; Shell – 1814; Cloud – 538.

**
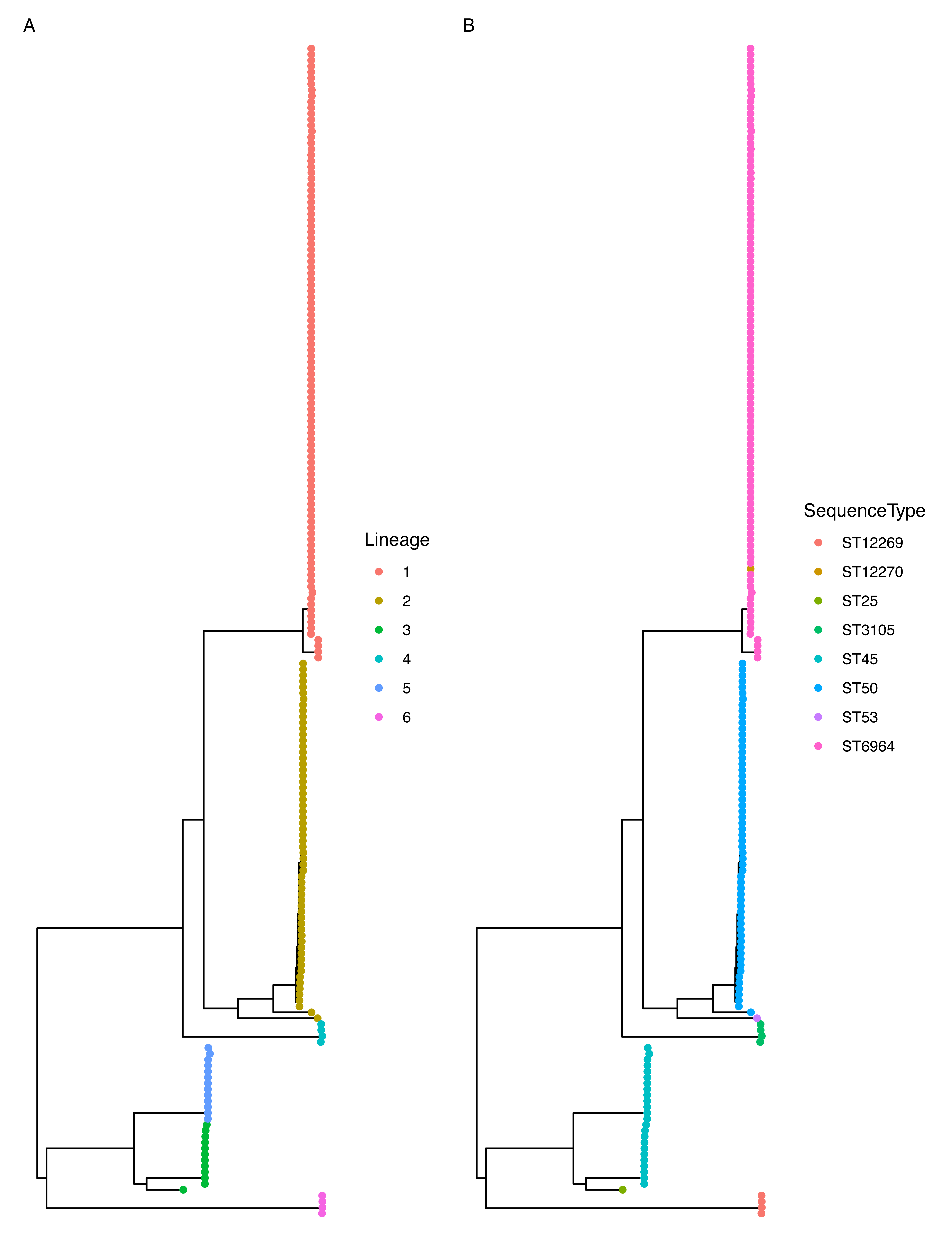
**

**Supplementary Figure 4.** Phylogeny showing (A) lineages from the rhierBAPS analysis and (B) STs, using the same tree. From the core 984 genes, 717 were analysed that fulfilled the criteria of having computed Tajima's D value of either => 2 or <= -2. The computed tree shows that there are six lineages (A) across the eight STs (B). The ST45 isolates split into two lineages, and the ST50 isolates and single ST53 join to become a single lineage.


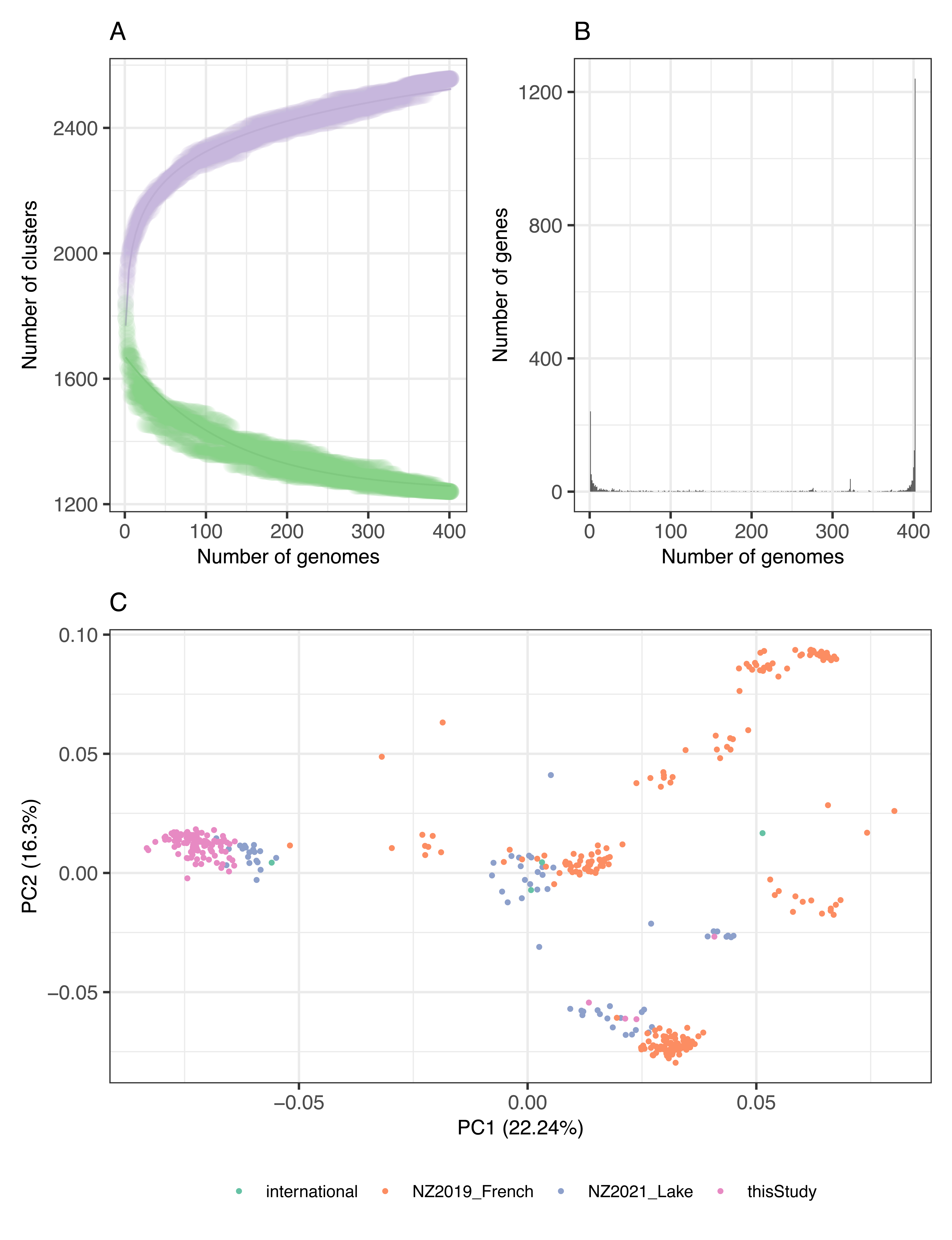


**Supplementary Figure 5.** Summary of the pangenome analyses of the 104 ST6964 isolates from this study, and three other studies. A) Plots of core genome and pangenome for the 402 isolates (Core – 1240; Shell – 1076; Cloud – 241; Total – 2257). B)  Barplot with the frequency of genes within the total number of genomes. C) Principal component analysis (PCA) of the isolates coloured by study. Other isolates were sourced from New Zealand poultry carcasses and human cases of campylobacteriosis from 2014-2016 (230 isolates designated NZ2019_French (2)) and from 2019 (64 isolates designated NZ2021_Lake (3)), as well as four isolates from other countries (designated “international”).


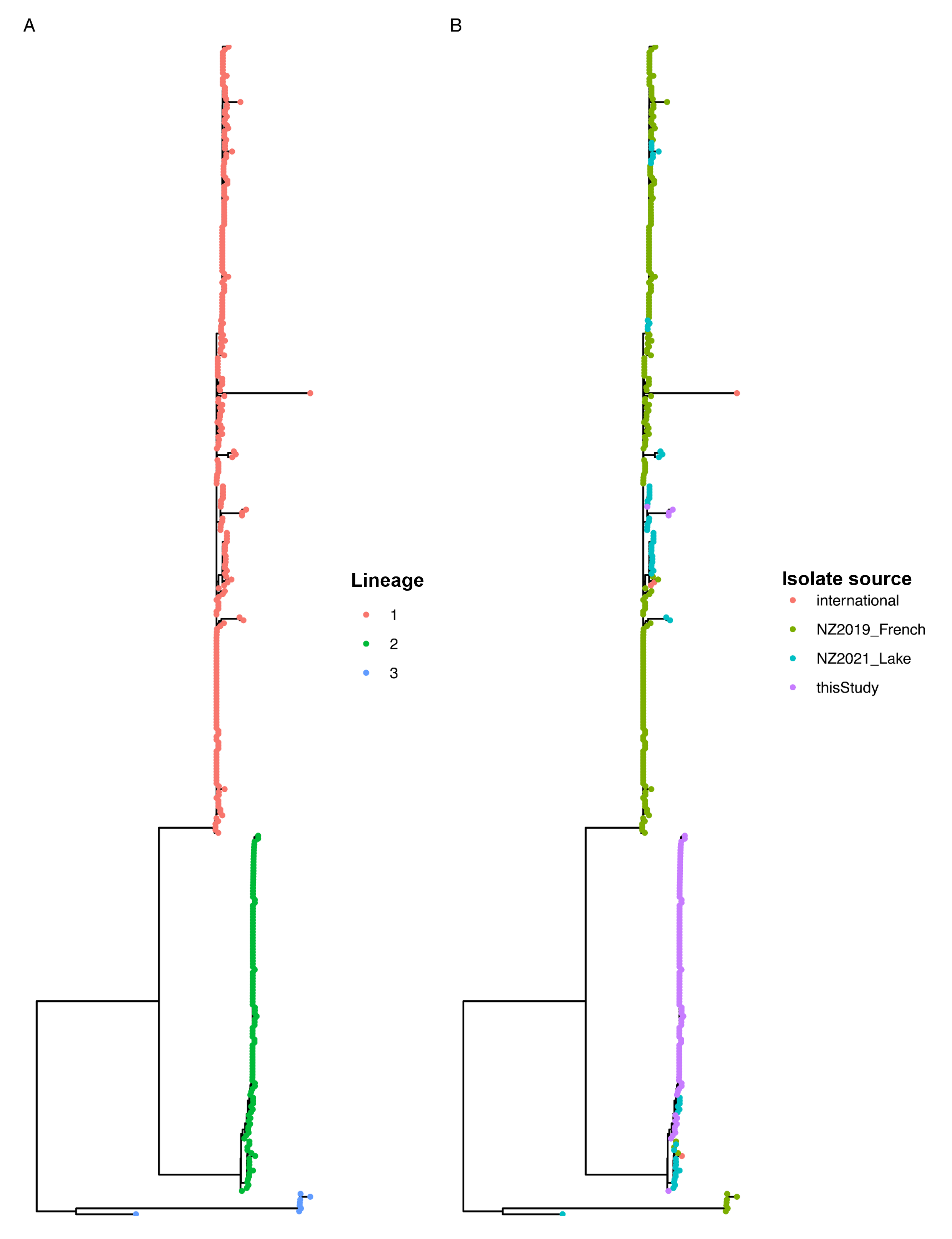


**Supplementary Figure 6.** Phylogeny of C. jejuni ST6964 isolates (A) showing lineages from rhierBAPS analysis and (B) isolate source, using the same tree. Isolates were sourced from this study (104 isolates), New Zealand poultry carcasses and human cases of campylobacteriosis from 2014-2016 (230 isolates designated NZ2019_French (2)) and from 2019 (64 isolates designated NZ2021_Lake (3)), as well as four isolates from other countries (designated “international”). From the core 1240 genes, 193 were analysed that fulfilled the criteria of having computed Tajima's D value of either => 2 or <= -2.

**Supplementary methods and results**

**Laboratory limit-of-detection pilot study to test suitability of sample types and testing methodology**

***Methods***

**Sample inoculation.** Prior to commencement of the farm survey, a pilot study tested the suitability and limit of detection of proposed test methods for a selection of sample types: elasticized hair covers in MRD proposed for use as boot socks, 3M^TM^ Enviro-swabs, houseflies with and without flypaper, and unused litter consisting of wood shavings (with and without MRD). These sample types were selected because they were not routinely tested by the Tegel New Plymouth laboratory, were diverse, or might contain competing microflora or inhibitors that affect downstream analyses. Sample types are described in Supplementary Table 6.

**Supplementary Table 6**: Sample description, preparation and testing.

| **Sample type** | **Sample description** | **Sample testing** |
| --- | --- | --- |
| Boot sock pair | - 1 pair of boot socks per test unit. - Boot socks comprised disposable, elasticised hair covers (Med-X round bouffant, Fabri-cell). - Boot socks were autoclaved, then pre-moistened with 20 ml Maximum Recovery Diluent (MRD) by the testing laboratory and supplied to the organizing laboratory. - A 1 ml volume of inoculum was applied to both boot socks by testing laboratory at multiple sites. - Inoculated samples were submitted to the testing laboratory in a Fisherbrand sampling bag. | 200 ml Bolton broth was added to boot sock pairs and stomached for 2 min prior to enrichment. |
| 3M^TM^ Enviro-swab | - 1 x 3M^TM^ Enviro-swab (ENVSWB100) per test unit. - Enviro-swabs were pre-moistened by the manufacturer in neutralizing solution. - Swabs were inoculated with 1 ml inoculum. - Inoculated swabs were submitted to the testing laboratory in transport tube supplied with swab. | Flexi-swabs were removed from the applicator stick and added to a 30 ml pot containing 20 ml Bolton broth. Applicator stick container was rinsed with a portion of the Bolton broth to recover any remaining sample. |
| Houseflies | - Recently pupated houseflies (Animates, Christchurch, New Zealand) were euthanized by placing in the freezer and stored there prior to inoculation.   *Experiment 1:*   - 10 houseflies in 10 ml MRD per test unit. - Houseflies were transferred to Fisherbrand sample bags using sterile forceps. Houseflies were shaken down to a corner of the bag and a 100 µl inoculum volume was applied to all ten flies externally. The lower inoculum volume of 100 µl was due to the small size of houseflies relative to the other types of samples. MRD was added 30-45 min following inoculation (to allow inoculum to dry/adsorb onto flies). - Inoculated flies were submitted to the testing laboratory in a Fisherbrand sampling bag (14 x 22 cm).   *Experiment 2:*   - 10 houseflies on 5 cm length of flypaper (Number 8 brand, “poison-free”, Mitre 10, New Zealand;) with 10 ml MRD per test unit. - 10 houseflies were transferred onto flypaper (~4-5 cm strip, cut with ethanol-sterilised scissors) on petri dishes (one dish per inoculum level). Flies on flypaper were each inoculated with ~10 µl volumes of inoculum (100 µL per test unit). - The inoculum was left to dry for 30 min and flies together with flypaper were placed in a Whirl-Pak sampling bag (17 x 28 cm) to which 10 ml MRD had been added. | Houseflies were macerated within the Fisherbrand bag and MRD buffer using three rolls of a rolling pin. A 90 ml volume of Bolton broth was added to the flies. |
| Litter | - 10 g per test unit. - Fresh, unused wood shavings were obtained from a bulk supply store. - Litter was inoculated at different points with a 1 ml volume of inoculum.   *Experiment 1:*   - Inoculated litter was supplied to the testing laboratory in a Fisherbrand sampling bag (14 x 22 cm).   *Experiment 2:*   - 20 ml MRD was added to 10 g litter 3 hours prior to inoculation. - Inoculated litter was supplied to the testing laboratory in a Whirl-Pak sampling bag (17 x 28 cm). | *Experiment 1:* 90 ml Bolton broth added to litter and stomached for 2 min prior to enrichment.  *Experiment 2:* 170 ml Bolton broth added to litter and stomached for 2 min prior to enrichment. |

At the organizing laboratory (Institute of Environmental Science and Research (ESR), Christchurch, New Zealand), *Campylobacter* inoculum cocktail of 12 poultry-relevant or reference *C. coli* and *C. jejuni* strains (Supplementary Table 7) was prepared from strains cultured on Columbia Blood Agar (CBA) plates, as shown in Supplementary Figure 7. A suspension of each strain was individually prepared in 3 ml Phosphate Buffered Saline (PBS) by swabbing colonies from CBA plates. The optical density (OD_600nm_) of the PBS/*Campylobacter* suspensions was adjusted to 0.4 (equivalent to ~10^8^ CFU/ml, as determined by plating). Volumes of 1 ml of each suspension were combined into a separate tube (~10^8^ CFU/ml), and a ten-fold serial dilution series was prepared in PBS. The 10^-5^ and 10^-6^ dilutions (containing ~10^3^ and ~10^2^ CFU/ml, respectively) were plated onto CBA plates for enumeration (1 ml spread over three plates, in duplicate), and these plates were incubated at 37˚C for 48 hours under microaerophilic conditions (was obtained via a CampyGen^TM^ Atmosphere Generation System; Oxoid, Hampshire, UK) to determine actual inoculum levels.

Samples were inoculated in triplicate with different concentrations of the cocktail. For the first experiment, samples were inoculated with a target inoculum cocktail concentration of 0 (PBS only), 10, 100 or 1000 colony forming units (CFU) per test unit. A second experiment was undertaken for the houseflies and litter samples because modifications to testing were deemed necessary. For the second experiment, litter and houseflies on flypaper were inoculated with target inoculum concentrations of 10, 100 and 1000 CFU per test unit. Because *Campylobacter* was not detected on the samples inoculated with 0 CFU in the first experiment, this control was not included in the second experiment. Larger Whirl-Pak sampling bags were used for both sample types for the second experiment (17 x 28 cm compared with 14 x 22 cm) because the bags used in the first experiment were not sufficiently large for adequate stomaching or macerating of samples.

**Supplementary Table 7**. *Campylobacter* strains used in the limit of detection study

| **Species** | **Strain/Isolate** | **Sequence type (ST)** | **Isolation source** |
| --- | --- | --- | --- |
| *C. jejuni* | ATCC 33560 (NZRM 2397) | ST403 | Bovine feces, laboratory type strain |
|  | P1566e | ST6964 | Chicken carcass |
|  | P1597a | ST48 | Chicken carcass |
|  | P1570c | ST45 | Chicken carcass |
|  | P1485a | ST53 | Chicken carcass |
|  | P1582b | ST583 | Chicken carcass |
|  | P1539a | ST50 | Chicken carcass |
|  | P1593d | ST2345 | Chicken carcass |
|  | P1569b | ST474 | Chicken carcass |
| *C. coli* | ATCC 33559 (NZRM 2607) | ST900 | Pig feces, laboratory type strain |
|  | P1572b | ST2256 | Chicken carcass |
|  | P1581a | ST1581 | Chicken carcass |


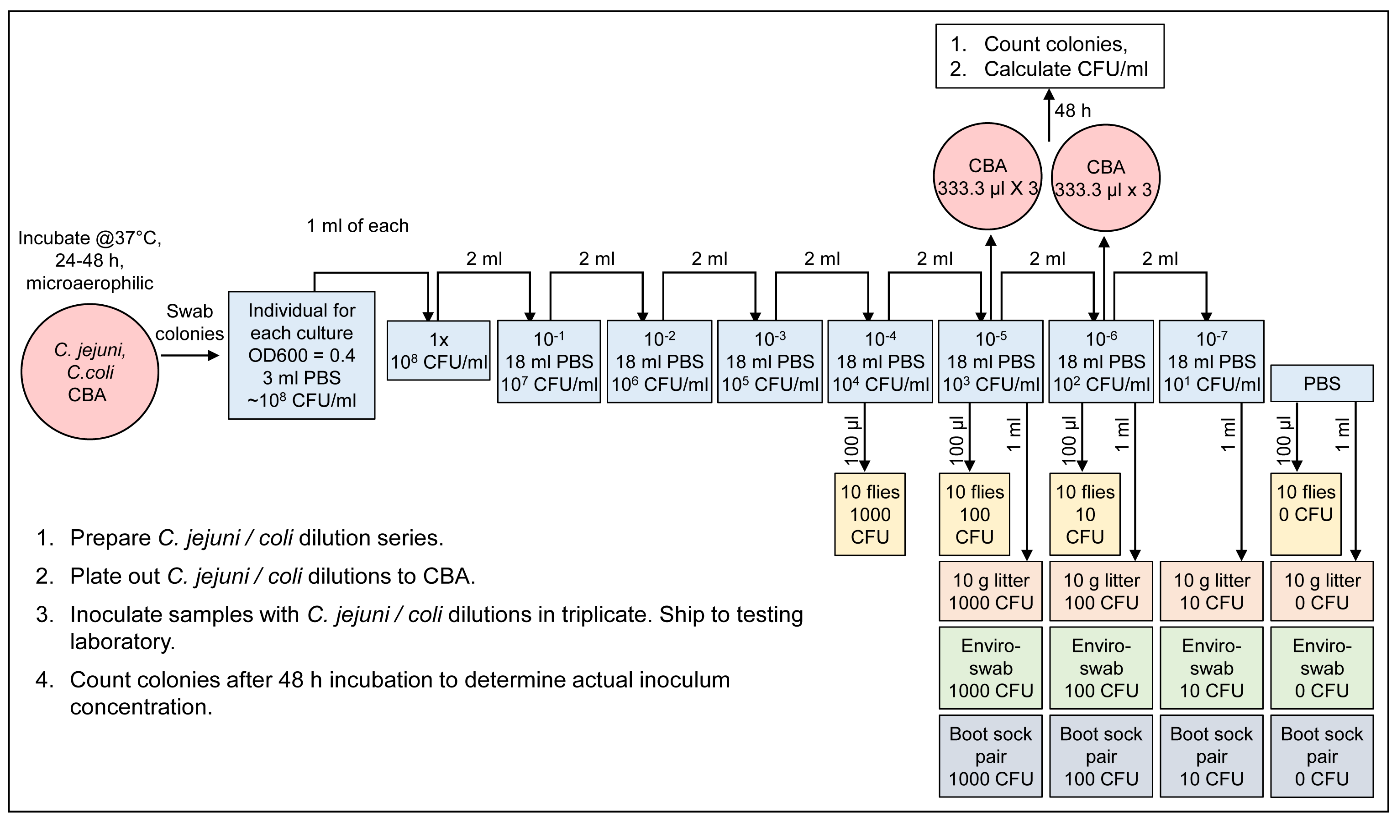


**Supplementary Figure 7**. Schematic for preparation of *Campylobacter* inoculum and inoculation of poultry samples.

**Sample testing.** Samples were couriered overnight on ice packs (~4°C) to the testing laboratory (Tegel New Plymouth, New Zealand). Sample testing commenced on the day of receipt, one day following sample inoculation. Samples were processed and tested as described in Supplementary Table 3. All sample enrichments were incubated in a sealed jar with a microaerophilic atmosphere provided using CampyGen sachets (Oxoid, Hampshire, UK). Samples were incubated at 35˚C for 4 hours followed by 42˚C for 44 hours. Following enrichment, a 10 µl volume of the enrichment culture was streaked onto mCCDA plates. Plates were incubated at 42°C and examined after 48 ± 2 hours for the presence of suspect *Campylobacter* colonies. Individual suspect colonies were streaked onto CBA plates and incubated at 42˚C. Isolates were confirmed as *Campylobacter* spp. via *Campylobacter* latex agglutination (Ngaio *Campylobacter* Latex, Ngaio Diagnostics, Nelson, NZ) and by detection of oxidase activity (Microbact oxidase strips, Oxoid, Hampshire, UK). The positive control strain *C. jejuni* NZRM 1958 and negative control strain *E. coli* NZRM 916 were included as media controls.

Two purified *Campylobacter* spp. isolates for each positive sample were swabbed onto Amies Transport Medium with charcoal (Copan, Murrieta, CA, USA) and sent on ice to mEpiLab, Massey University, New Zealand. The species was determined using MALDI-TOF MS with full protein extraction in a Bruker Biotype (4).

**Calculation of LOD_50_ and statistical analyses.** The limit of detection at 50% (LOD_50_) is the concentration of *Campylobacter* (CFU/test portion) for which the probability of detection is 50%. LOD_50_ calculations require a fractional recovery at the lowest inoculum level as a minimum. LOD_50_ values and 95% confidence intervals were calculated as described previously, using an Excel calculator available from the author’s website (5).^^[[8]](#footnote-9)^^ Because the method involved taking the log of the concentration values, a value of 0.004 (instead of 0) was used for uninoculated controls.^[[9]](#footnote-10)^

***Results and discussion***

The results for *Campylobacter* detection (presence/absence) and species isolated (*C. jejuni* or *C. coli*) for each sample are provided in Supplementary Table 8.

Of the sample types tested, the best recovery was from houseflies, in the presence or absence of flypaper. Having a detection method that is as-sensitive-as-possible is particularly important for isolating *Campylobacter* from samples where the concentration may be low, such as flies. Therefore, our results support the use of the sampling and testing methods used, including flypaper for the capture of flies and its inclusion in the enrichment broth. An advantage of testing the flypaper together with the flies is that flypaper has the potential to capture very small flying insects which might be difficult to remove from the flypaper, but yet might be relevant vectors. In addition, *Campylobacter* present on flies could be adhering to fly legs, which might become detached during the retrieval of flies from the flypaper. A previous study reported the presence of inhibitors in glue traps (6); therefore, it will be important to use the same brand demonstrated here to be non-inhibitory (Number 8 brand). The same study also reported excessive drying out of flies on glue traps, which inhibits *Campylobacter* survival (6). As such, flypapers should be hung no longer than 48 hours prior to sample collection. The addition of MRD to samples, either immediately after sample collection or following receipt at the laboratory, would also be pertinent.

The levels of detection of *Campylobacter* from boot sock pairs (designed to be used as hair covers) and Enviro-swabs were similar, with LOD_50_ of 37.9 (9.2-156.8) and 47.2 (10.7-207.9) CFU per test unit, respectively. Therefore, although the hair covers were not specifically designed for the purpose of sampling, they were proven suitable, and are listed by ISO 13307:2013 as an acceptable boot sock option (7). It is anticipated that the LOD_50_ values for swabs and boot socks reported here will be the best-case scenario for these sample types (assuming testing on the day following sampling) because actual poultry samples will likely contain competing background microflora. Boot socks are used for the capture of feces and cecal droppings, as well as other contaminants in the litter. Because *Campylobacter* is typically present in high concentrations in the feces or cecal contents (10^4^ to >10^6^ CFU/g) of colonised chickens (8, 9), the observed LOD_50_ of ~38 CFU/test unit for boot socks in this study should be more than adequate for sampling purposes.

*Campylobacter* were not recovered from dry litter samples, and were only recovered from samples inoculated at the highest concentrations and with MRD buffer added. Previously, *Campylobacter* was demonstrated to rapidly lose viability in dry litter (e.g. a reduction of 2-5 log_10_ CFU/g over a 12-hour period depending on the environmental conditions) (10), and the addition of buffers such as MRD have been recommended to improve *Campylobacter* survival in other dry sample types (7). The poor survival and recovery of *Campylobacter* from litter even in the presence of MRD might also be due to the presence of naturally occurring essential oils present in the litter, such as pinosylvans and phenolic compounds (e.g. lignin), which have demonstrated antimicrobial activity against various microbes including *Campylobacter* (7, 11, 12). Despite the low recovery of *Campylobacter* from litter, even a low level of contamination of clean litter might be relevant given the large volume applied to the broiler shed. Therefore, litter sampling was still included in the survey.

Taken together, the sampling and testing methods established and refined by this study were suitable for inclusion in broiler farm survey. In addition, the LOD results obtained informed on the results that might be expected from equivalent sample types of *Campylobacter* isolation from these samples.

**Supplementary Table 8.** *Campylobacter* detection from inoculated poultry samples.

| **Sample type** | **CFU/**  **test unit** | **Replicate 1** | **Replicate 2** | **Replicate 3** | **Total** | |
| --- | --- | --- | --- | --- | --- | --- |
|  |  | **Species^1^** | **Species^1^** | **Species^1^** | **Prevalence** | **Species^1^** |
| Experiment 1 | | | | | | |
| Boot sock pair/MRD | 0 | ND^5^ | ND | ND | 0/3 | ND |
|  | 5.1 | ND | ND | ND | 0/3 | ND |
|  | 51 | *C. jejuni* (2) | ND | *C. coli* (2) | 2/3 | *C. coli* (2),  *C. jejuni* (2) |
|  | 510 | *C. coli* (2) | *C. coli* (2) | *C. coli* (2) | 3/3 | *C. coli* (6) |
| LOD_50_ | 37.9 (9.2-156.8) CFU per test unit | | | Total | 5/12 | *C. coli* (8),  *C. jejuni* (2) |
| Enviro-swab | 0 | ND | ND | ND | 0/3 | ND |
|  | 5.1 | *C. coli* (2) | ND | ND | 1/3 | *C. coli* (2) |
|  | 51 | *C. jejuni* (2) | ND | ND | 1/3 | *C. jejuni* (2) |
|  | 510 | *C. coli* (2) | *C. coli* (2) | *C. coli* (2) | 3/3 | *C. coli* (6) |
| LOD_50_ | 47.2 (10.7-207.9) CFU per test unit | | | Total | 5/12 | *C. coli* (8),  *C. jejuni* (2) |
| Houseflies/MRD | 0 | ND | ND | ND | 0/3 | ND |
|  | 5.1 | *C. jejuni* (2) | ND | ND | 1/3 | *C. jejuni* (2) |
|  | 51 | *C. coli* (2) | *C. coli* (2) | *C. coli* (2) | 3/3 | *C. coli* (6) |
|  | 510 | *C. coli* (2) | *C. coli* (2) | *C. coli* (2) | 3/3 | *C. coli* (6) |
| LOD_50_ | 7.7 (1.7-35.0) CFU per test unit | | | Total | 7/12 | *C. coli* (12),  *C. jejuni* (2) |
| Litter | 0 | ND | ND | ND | 0/3 | ND |
|  | 5.1 | ND | ND | ND | 0/3 | ND |
|  | 51 | ND | ND | ND | 0/3 | ND |
|  | 510 | ND | ND | ND | 0/3 | ND |
| LOD_50_ | >989.9 (135.0-7,261.6) CFU per test unit^3^ | | | Total | 0/12 | ND |
|  |  | | | Experiment 1 total | 17/48 | *C. coli* (28),  *C. jejuni* (6) |
| Experiment 2 | | | | | | |
| Houseflies on flypaper/  MRD | 33 | *C. jejuni* (2) | *C. jejuni* (2) | *C. jejuni (1), C. coli (1)* | 3/3 | *C. coli* (1),  *C. jejuni* (5) |
|  | 330 | *C. jejuni (1), C. coli (1)* | *C. coli (1)* | *C. coli* (2) | 3/3 | *C. coli* (4),  *C. jejuni* (1) |
|  | 3,300 | *C. coli* (2) | Not tested | | 1/1^2^ | *C. coli* (2) |
| LOD_50_ | <20.8 (4.7-91.8) CFU per test unit^4^ | | | Total | 7/7 | *C. coli* (7),  *C. jejuni* (6) |
| Litter/MRD | 33 | ND | ND | ND | 0/3 | ND |
|  | 330 | ND | ND | ND | 0/3 | ND |
|  | 3,300 | *C. jejuni* (2) | *C. coli* (2) | ND | 2/3 | *C. coli* (2),  *C. jejuni* (2) |
| LOD_50_ | 2,492.3 (599.6-10,359.0) CFU per test unit | | | Total | 2/9 | *C. coli* (2),  *C. jejuni* (2) |
|  |  | | | Experiment 2 total | 9/16 | *C. coli* (9),  *C. jejuni* (8) |

^1^ Up to two isolates per positive sample were typed by MALDI-TOF MS analysis; ND not detected.

^2^ Due to insufficient houseflies, only one sample was tested at the highest inoculum level (3,300 CFU/test unit), and this sample only contained 5 instead of 10 flies.
^3^ Because all samples tested negative, the LOD_50_ was calculated based on one sample at the highest inoculum level testing positive.

^4^ Because all samples tested positive, the LOD_50_ was calculated based on one sample at the lowest inoculum level testing negative.

**REFERENCES**

1. Ministry for Primary Industries. 2023. Animal Products Notice: National Microbiological Database Programme. Ministry for Primary Industries, Wellington, New Zealand.

2. French NP, Zhang J, Carter GP, Midwinter AC, Biggs PJ, Dyet K, Gilpin BJ, Ingle DJ, Mulqueen K, Rogers LE, Wilkinson DA, Greening SS, Muellner P, Fayaz A, Williamson DA. 2019. Genomic analysis of fluoroquinolone- and tetracycline-resistant *Campylobacter jejuni* sequence type 6964 in humans and poultry, New Zealand, 2014-2016. Emerging Infectious Diseases 25:2226-2234.

3. Lake RJ, Campbell DM, Hathaway SC, Ashmore E, Cressey PJ, Horn BJ, Pirikahu S, Sherwood JM, Baker MG, Shoemack P, Benschop J, Marshall JC, Midwinter AC, Wilkinson DA, French NP. 2021. Source attributed case-control study of campylobacteriosis in New Zealand. International Journal of Infectious Diseases 103:268-277.

4. Nisa S, Bercker C, Midwinter AC, Bruce I, Graham CF, Venter P, Bell A, French NP, Benschop J, Bailey KM, Wilkinson DA. 2019. Combining MALDI-TOF and genomics in the study of methicillin resistant and multidrug resistant *Staphylococcus pseudintermedius* in New Zealand. Scientific Reports 9:1271.

5. Wilrich C, Wilrich PT. 2009. Estimation of the POD function and the LOD of a qualitative microbiological measurement method. Journal of AOAC International 92:1763-72.

6. Hald B, Skovgård H, Pedersen K, Bunkenborg H. 2008. Influxed insects as vectors for *Campylobacter jejuni* and *Campylobacter coli* in Danish broiler houses. Poultry Science 87:1428-1434.

7. International Organization for Standardization. 2013. ISO 13307:2013 Microbiology of food and animal feed -- Primary production stage -- Sampling techniques. International Organization for Standardization, Geneva, Switzerland.

8. International Organization for Standardization. 2017. ISO 10272-1. Microbiology of the food chain — Horizontal method for detection and enumeration of *Campylobacter* spp. —Part 1: Detection method. International Organization for Standardization, Switzerland.

9. Anonymous. 2015. Comparison of sample types and analytical methods for the detection of highly *Campylobacter*-colonized broiler flocks at different stages in the poultry meat production chain. Foodborne Pathogens and Disease 12:399-405.

10. Smith S, Meade J, Gibbons J, McGill K, Bolton D, Whyte P. 2016. The impact of environmental conditions on *Campylobacter jejuni* survival in broiler faeces and litter. Infection Ecology & Epidemiology 6:31685-31685.

11. Kurekci C, Padmanabha J, Bishop-Hurley SL, Hassan E, Al Jassim RAM, McSweeney CS. 2013. Antimicrobial activity of essential oils and five terpenoid compounds against *Campylobacter jejuni* in pure and mixed culture experiments. International Journal of Food Microbiology 166:450-457.

12. Zeng W-C, Zhang Z, Gao H, Jia L-R, He Q. 2012. Chemical composition, antioxidant, and antimicrobial activities of essential oil from pine needle (*Cedrus deodara*). Journal of Food Science 77:C824-C829.

1. This data was not collected on-farm, conditions for the area were accessible from NIWA. [↑](#footnote-ref-2)
2. Samples within a sample set were sampled on the same dates. [↑](#footnote-ref-3)
3. For the first sampling event, boot socks comprised sterilized overshoe safety covers (NZ Safety Blackwoods) in the absence of MRD. [↑](#footnote-ref-4)
4. The ethics approval allowed for a maximum of 480 chickens to be sampled. [↑](#footnote-ref-5)
5. Ethics approval did not cover direct sampling of workers. No worker samples identified the worker and were collected from clothing following removal from worker. [↑](#footnote-ref-6)
6. [↑](#footnote-ref-7)
7. Feed samples were collected from chicken age 20 days onward. [↑](#footnote-ref-8)
8. <https://www.wiwiss.fu-berlin.de/fachbereich/vwl/iso/ehemalige/wilrich>; accessed 8 July 2024 [↑](#footnote-ref-9)
9. <https://www.fda.gov/media/73476/download>; accessed 9 July 2024 [↑](#footnote-ref-10)
